# Replay and representation dynamics in the hippocampus of freely flying bats

**DOI:** 10.1038/s41586-025-09341-z

**Published:** 2025-07-09

**Authors:** Angelo Forli, Wudi Fan, Kevin K. Qi, Michael M. Yartsev

**Affiliations:** 1 Department of Bioengineering, University of California, Berkeley, Berkeley, CA USA; 2 Helen Wills Neuroscience Institute, University of California, Berkeley, Berkeley, CA USA; 3 Biophysics Graduate Group, University of California, Berkeley, Berkeley, CA USA; 4 Department of Neuroscience, University of California, Berkeley, Berkeley, CA USA; 5 Howard Hughes Medical Institute, University of California, Berkeley, Berkeley, CA USA

**Keywords:** Learning and memory, Neural circuits

## Abstract

Cognitive functions for navigation and memory rely on emergent properties of neural ensembles in the hippocampus, such as activity replay^1–5^ and theta sequences^6–9^. However, whether and how these phenomena generalize across species with distinct navigational demands and neurophysiological properties remains unclear. Here we wirelessly recorded neural activity from large populations of cells and local field potentials from the hippocampus of freely flying Egyptian fruit bats (*Rousettus aegyptiacus*) engaged in free, spontaneous foraging behaviour. During rest, we identified time-compressed forward and reverse replays of multiple flight trajectories coinciding with sharp-wave ripples. Notably, replays occurred predominantly at locations that were both spatially and temporally distant from the replayed behaviour, and their speed scaled with trajectory length, challenging present models of replay mechanisms. During flight, neural ensembles exhibited fast representational sweeps, in which the decoded location moved ahead of the bat’s position cyclically. In contrast to reports in rodents, sweeps occurred in the absence of theta oscillations, and were instead phase locked to a prominent motor behavioural rhythm—the bat’s wing-beat cycle. This suggests that behaviourally relevant sensorimotor rhythms can interact with hippocampal ensemble dynamics in a highly structured manner. Combined, our findings challenge existing models of ensemble dynamics in the mammalian hippocampus, and highlight the importance of comparative studies in ethologically relevant conditions for elucidating brain function.

## Abstract (web summary)

Wireless large-scale recordings of neural activity in freely flying fruit bats reveal that hippocampal ensemble properties are associated with sensorimotor rhythms, and that previous models of replay and sequential dynamics, based on experiments in rodents, might not be applicable to other species.

## Main

The spatial and temporal organization of experiences is crucial for forming memories of past events and for guiding future behaviours. Previous studies indicate that time-compressed and sequential ensemble phenomena in the hippocampus, such as replay^1–5^ and oscillatory representation dynamics (theta sequences)^6–9^, are central for supporting this process. However, nearly all previous investigations of these phenomena have been performed in rodents, often engaged in guided tasks. This raises two major challenges. First, because in guided tasks, movement and rest can be experimentally segregated and the spatial repertoire can be experimentally constrained (for example, on linear tracks or mazes), it remains unclear whether and how these phenomena extend to naturalistic behaviours—where spatial experiences are continuous, unconstrained and inherently variable, such that animals are free to choose when and where to move and rest. Second, in rodents, both replay and theta sequences—which are believed to be causally related^7,8,10^—are fundamentally tied to the presence of strong hippocampal theta oscillations during locomotion. Yet many species, including bats^11^ and primates^12^, lack continuous locomotion-related theta rhythms. This raises questions about the mechanisms that underlie these ensemble dynamics and whether they represent a universal feature of hippocampal computation, or instead reflect rodent-specific adaptations. To address these gaps, here we established large-scale wireless neural recordings in freely flying Egyptian fruit bats (*R*.* aegyptiacus*), and exploited their natural tendency to organize spontaneous foraging behaviour in a structured manner^13,14^, during both movement and rest. This allowed us to investigate ensemble dynamics through a naturally unfolding but highly controlled form of spatial behaviour.

## Hippocampal replay of flight trajectories

**Fig. 1:**
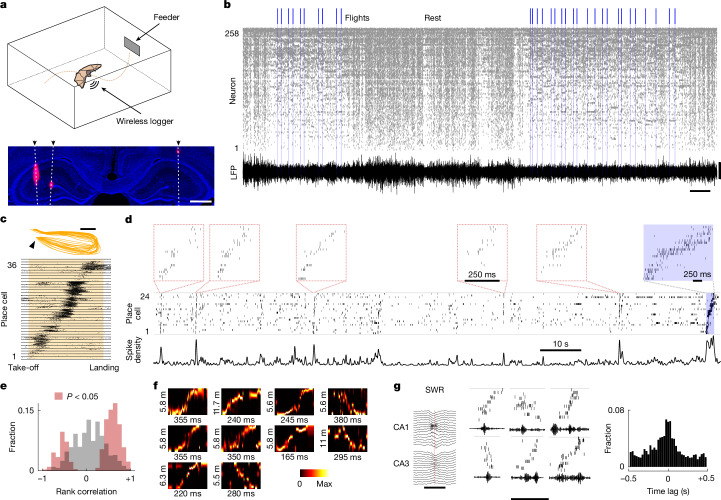
Wireless recordings with Neuropixels probes during aerial foraging reveal hippocampal replay in bats. **a**, Top, schematic of the aerial foraging experiment. Bottom, dorsal hippocampus (coronal section) in one recorded bat, stained for 4′,6-diamidino-2-phenylindole (DAPI, blue) and CM-DiI (red) (Methods). White dashed lines marked with arrowheads denote tracks of three implanted Neuropixels 1.0 probes. Scale bar, 1 mm. **b**, Raster plot of simultaneously recorded hippocampal neurons during a representative session. Neurons are sorted by average firing frequency. Bottom trace, average hippocampal LFP across all channels of one probe during the same session. Scale bars, 800 μV (vertical) and 1 min (horizontal). **c**, Top, similar flights from a representative session. Arrow indicates take-off. Bottom, raster plot of simultaneously recorded place cells, sorted by the location of their firing field (Methods). Each row shows the firing during 22 consecutive repetitions of the same trajectory. Neural activity is plotted along a normalized flight trajectory, with all flights temporally aligned and rescaled (yellow shaded area) such that take-off and landing coincide in the visualization. Scale bar, 2 m. **d**, Neural activation of place cells during a representative epoch. Top, raster plots showing sorted neural activity during time-compressed candidate replays (red rectangles) and during a flight (blue shaded area on the right). Note the different temporal scales. Neurons are sorted by the location of their firing fields. Middle, raster plot from the same neurons, showing their activity during the entire epoch. Bottom, spike density from the same neurons. **e**, Distribution of rank-correlation values for all candidate replay events (*n* = 16,468, from 23 sessions and 6 bats; Methods). Grey distribution indicates non-significant events and red distribution significant events (*P* value from rank-correlation analysis; Methods). **f**, Decoded probability of linearized position (take-off location is at the bottom; Methods) for example replay events. Numbers indicate the temporal and spatial scale of each replay. **g**, Left, example LFP across a subset of contacts (for visualization) during a representative SWR event. Scale bar, 100 ms. Middle, example raster plots (top) and LFP epochs in the pyramidal cell layer (bottom) during representative replays. Note the coincidence between replay and one or more SWRs. Scale bar, 350 ms. Right, average cross-correlogram between replay and SWR times (*n* = 23 sessions from 6 bats). Illustration in **a** adapted from ref. ^14^, Springer Nature Limited, under a Creative Commons licence CC BY 4.0. Source Data

**Extended Data Fig. 1:**
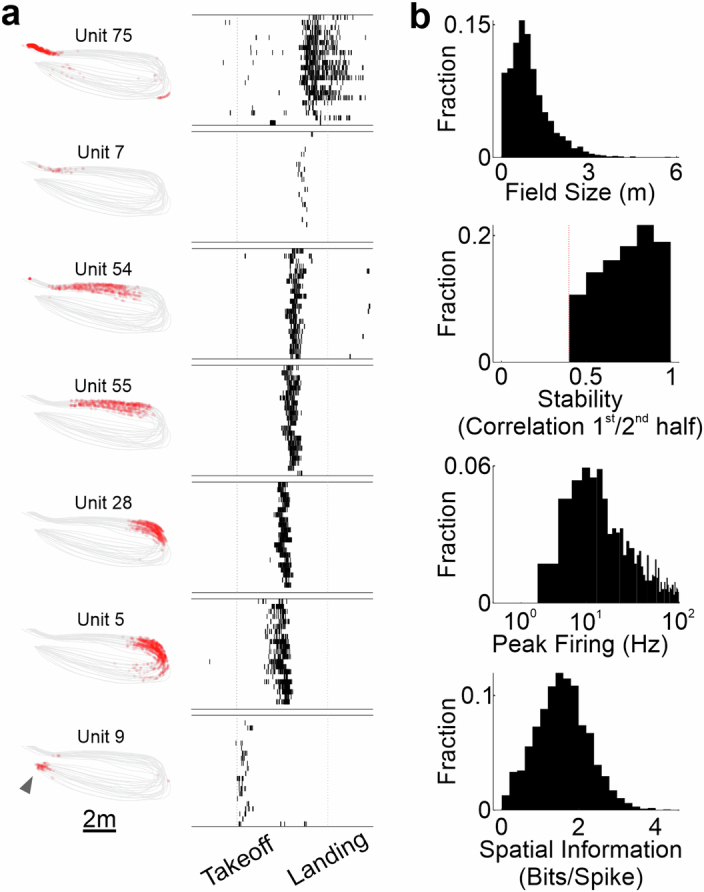
Spatially selective cells during flight. **a**, Example spatially selective cells across one flight trajectory. Left column depicts the flight trajectory (grey lines, top view) and overlaid spikes (red dots) of seven example neurons, sorted by location of peak firing from take-off (grey triangle) to landing (bottom to top row). Right column shows the raster plot across 22 repetitions of the same trajectory. Neural activity is plotted along a normalized flight trajectory, with all flights temporally aligned and rescaled, such that take-off and landing coincide in the visualization. Note that owing to the high similarity of flights, minimal rescaling is needed. **b**, Distributions of field size, stability, peak firing rate and spatial information (Methods) for 1,620 neurons x trajectory. Data are from 990 spatially tuned neurons, 6 bats (Methods).

**Extended Data Fig. 2:**
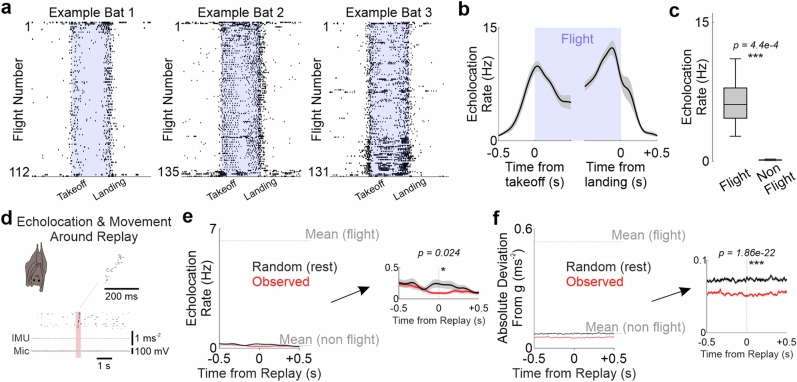
Echolocation rate and movement level around replay. **a**, Raster plots of echolocation calls (vertical ticks) from three different bats during individual flight sessions. Each flight epoch is normalized to have the same duration across trials. Non-flight epochs before and after each flight are shown with durations equal to the corresponding flight. **b**, Average echolocation rate around take-off and landing (*n* = 16 sessions from 4 bats). Shaded area represents s.e.m. **c**, Box plot for the average echolocation rate during flight versus non-flight (two-sided *P* from Wilcoxon signed-rank test, *n* = 16 sessions from 4 bats), The box plot shows the maximum and minimum values (whiskers), median (centre line) and 25th to 75th percentiles (box limits) across sessions. **d**, Schematics of the analysis of echolocation production and movement level around replay times. Mic: environment microphone; IMU: inertial measurement unit, measuring acceleration data on the headstage. **e**, Average echolocation rate around replay times (red trace) versus random epochs of non-flight (black trace, *n* = 2,364 replays from 16 sessions, 4 bats). Traces are compared with the time-averaged echolocation rate during flight and non-flight for the same sessions (dotted lines). Inset shows a magnified view of the plot; significance of the difference between the two traces is tested at time 0 (Wilcoxon signed-rank test, *n* = 2,364 replays from 16 sessions, 4 bats). **f**, Same as **e**, but for the movement around replay, quantified as the absolute value of the difference between the absolute acceleration and *g*. Note the near-zero echolocation production and minimal movement around replay times, which is even lower than during randomly selected non-flight epochs. In **e**,**f**, solid traces represent the mean and shaded areas are s.e.m. *P* values are two-sided.

**Extended Data Fig. 3:**
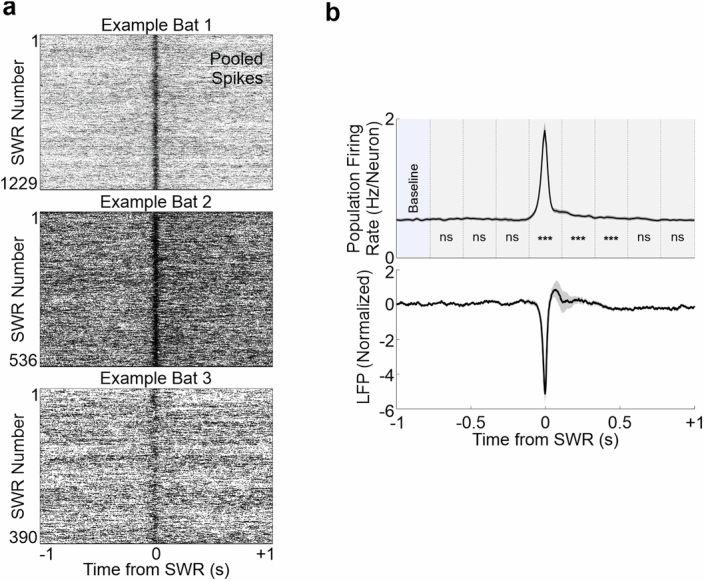
Population activity around SWRs. **a**, Raster plots from three example sessions (three different bats) of population activity around SWRs. Spikes (black ticks) are pooled from all the simultaneously recorded single units. **b**, Average population firing rate (top) and LFP profile (bottom, normalized between minimum and maximum) around SWRs. The average firing rate in each time bin (111 ms) is tested against baseline level (Wilcoxon signed-rank test, *n* = 22 sessions from 6 bats). Note that firing rate is indistinguishable from baseline up until the SWR event. Solid traces represent the mean and shaded areas are s.e.m. *P* values are two-sided. ***P < 0.001; ns: *P* > 0.05.

We used Neuropixels 1.0 probes^15^ to wirelessly record ensemble activity, at cellular resolution, from the dorsal hippocampus of Egyptian fruit bats engaged in rewarded spontaneous aerial foraging^13,16^ (*n* = 6 bats, 23 sessions; Fig. 1a). During recording sessions (mean duration: 1 h 20 min) we simultaneously monitored the activity of putative single neurons (49–322 per session) and the local field potential (LFP) while bats spontaneously alternated between periods of rest and flight (33–154 flights per session; Fig. 1b). Consistent with previous results in bats^13,14,16,17^, we found that a large portion of single units that were active during flight exhibited robust spatial selectivity (place cells: 990 out of 1,386 flight-active neurons (71%), *n* = 6 bats; Methods and Extended Data Fig. 1), spanning each flight trajectory from take-off to landing (Fig. 1c). We further observed that many of the same neurons that were active during flight were also transiently active during rest, and that they often covered a similar temporal sequence of activation, albeit in much shorter time windows (Fig. 1d). This was reminiscent of ‘replay’ events observed in the rodent hippocampus^3–5^. Because time-compressed sequential replay of single-unit activity in the hippocampus has thus far been characterized almost exclusively in rodents (but see ref. ^18^ for evidence of events involving triplets of neurons in humans), we first analysed the nature of putative replay events, detected as brief increases in the place-cell spike density during rest (bottom trace in Fig. 1d; Methods). For each candidate event, we calculated the rank correlation^4^ between the sequence of cell activations and time, along with its statistical significance (Methods). Numerous events had significant rank correlations and involved a considerable fraction of the flight-active cells (fraction of active cells: 0.45 ± 0.14 (median ± s.d.); *n* = 2,887 replays), the activity of which was compressed within a short time window (replay duration: 358 ± 185 ms (median ± s.d.); *n* = 2,887 replays). Events with positive correlation (forward replays) outnumbered those with negative correlation (reverse replays) under our experimental conditions (2,050 forward replays out of 2,887 total replays (71%); Fig. 1e). Replays occurred during epochs of low movement and in the absence of echolocation production (the latter being restricted mostly to flight epochs; Extended Data Fig. 2). As a complementary approach, we used a memory-less, uniform-prior Bayesian decoding algorithm^19^ to decode the position of the bat from neural activity throughout a session (Methods). This procedure allowed us to find candidate events in which the decoded position probability during rest epochs resembled the one during flight trajectories. Notably, the continuous-time decoding approach provided a robust and independent complement to the spike-sequence analysis, because it did not rely on predefined place-cell identification or spike-density segmentation and was inherently less influenced by spike-sorting caveats^20^. For each candidate event, we calculated a series of quality scores from the decoded probability and assessed their significance through shuffling analysis^20,21^ (Methods). This procedure again revealed the presence of replay-like episodes (Fig. 1f), with forward replay being more frequent than reverse replay (2,155 forward replays out of 3,775 total replays; 57%). We further examined the temporal relationship between replays and sharp-wave ripples (SWRs): brief and highly synchronous network oscillations with a characteristic LFP signature across the dorsal hippocampus^11,22^ (Fig. 1g, left) that were associated with a temporary increase in population firing rate (Extended Data Fig. 3). In agreement with previous findings in rodents^3–5^, replay events coincided with SWRs (Fig. 1g, middle), occurring within a short time interval from the SWR centre (Fig. 1g, right). These results show that replays—that is, time-compressed sequential activations of spatially selective neurons—are present in the bat hippocampus. Moreover, such replay events progress in both forward and reverse directions, and are associated with SWRs, consistent with previous reports in rodents^3–5^. Next, we investigated replay dynamics, using the natural diversity and complexity of bats’ spontaneous spatial behaviour.

## Replay dynamics during aerial foraging

**Fig. 2:**
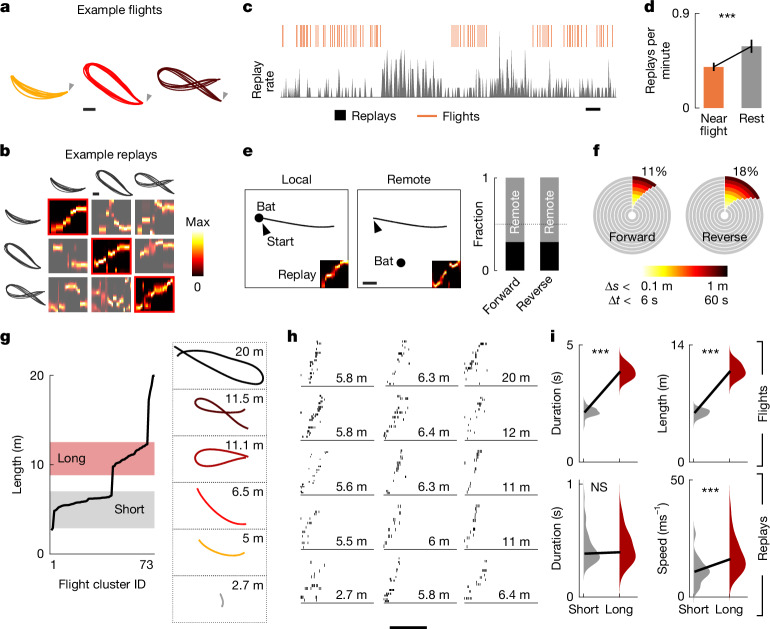
Replay dynamics across spatial behaviours during aerial foraging. **a**, Example flight trajectories (top view) from one bat during a representative session. Flights are clustered into similar paths (colours); arrow denotes take-off. Scale bar, 1 m. **b**, Example posterior probabilities (squares) for candidate replays decoded using place fields from different trajectories (traces). Note the decoding specificity of replays. Scale bar, 1 m. **c**, Normalized replay rate (grey) and flight times (orange lines) during a session. Scale bar, 5 min. **d**, Replay rate around flight times (orange, within 30 s of a flight) versus periods of rest (grey, more than 30 s away from a flight). Bars denote the average replay rate; vertical lines denote s.e.m. ***P = 9.7 × 10^−8^, two-sided Wilcoxon signed-rank test (*n* = 71 trajectories from 19 sessions across 5 bats). **e**, Left, example of a local replay. Black dot, position of bat at time of replay; arrow, take-off; black trace, average flight trajectory being replayed. Middle, same, but for a remote replay. Scale bar, 1 m. Right, bar plot for the median proportion of local and remote replays (*n* = 19 sessions, 5 bats). **f**, Pie charts showing the percentage of spatial and temporally local replays with varying thresholds (the replays shown in the chart meet both spatial and temporal proximity criteria simultaneously). Δ*s*, distance between location of bat at time of replay and start location of replay; Δ*t*, time interval between replay and take-off (forward) or landing (reverse) of nearest replayed trajectory (*n* = 2,050 forward replays, 837 reverse replays from 23 sessions and 6 bats). **g**, Left, cumulative distribution of flight trajectory lengths (73 trajectories, 23 sessions from 6 bats). Shaded coloured rectangles are the groups of short and long flights used in **i**. Right, example average trajectories (top view) for different lengths. **h**, Example raster plots for forward replays of trajectories with different lengths, sorted from bottom left to top right. Note the near constant duration of replays across lengths. Scale bar, 500 ms. **i**, Top, average flight duration (left) and length (right) for the group of short (*n* = 37) versus long (*n* = 28) flight clusters (see **g**). Two-sided *P* = 7 × 10^−12^ and *P* = 7 × 10^−12^, Wilcoxon rank-sum test. Bottom, average replay duration (left) and speed (right) for the same groups. Two-sided *P* = 0.796 and 9.56 × 10^−5^, Wilcoxon rank-sum test; NS, not significant (*n* = 37 versus *n* = 28 flight clusters). Thick line represents median, coloured area (violin) represents the data distribution across flight trajectories. Note that replay speed, but not duration, scales with flight length. Source Data

**Extended Data Fig. 4:**
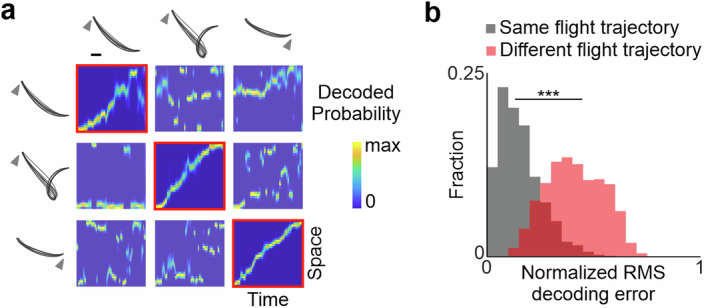
Different neural ensembles are active during different flights. **a**, Example posterior probability matrixes obtained by decoding neural activity during single flights, when using neural ensembles extracted from the same flight trajectory (diagonal) or from another flight trajectory (off-diagonal) during the same experimental session. Scale bar, 1 m. **b**, Root mean squared error (normalized; Methods) when decoding the bat’s position during flight using the ensemble of cells active during the same flight (grey, 1,479 flight trajectories from 23 sessions, 6 bats) or a different flight within the same session (red, 4,544 flight trajectory pairs from 23 sessions, 6 bats). two-sided *P* = 0, two-sample Kolmogorov–Smirnov test. Note that bats, unlike rodents, never traversed the same trajectory in two opposite directions.

**Extended Data Fig. 5:**
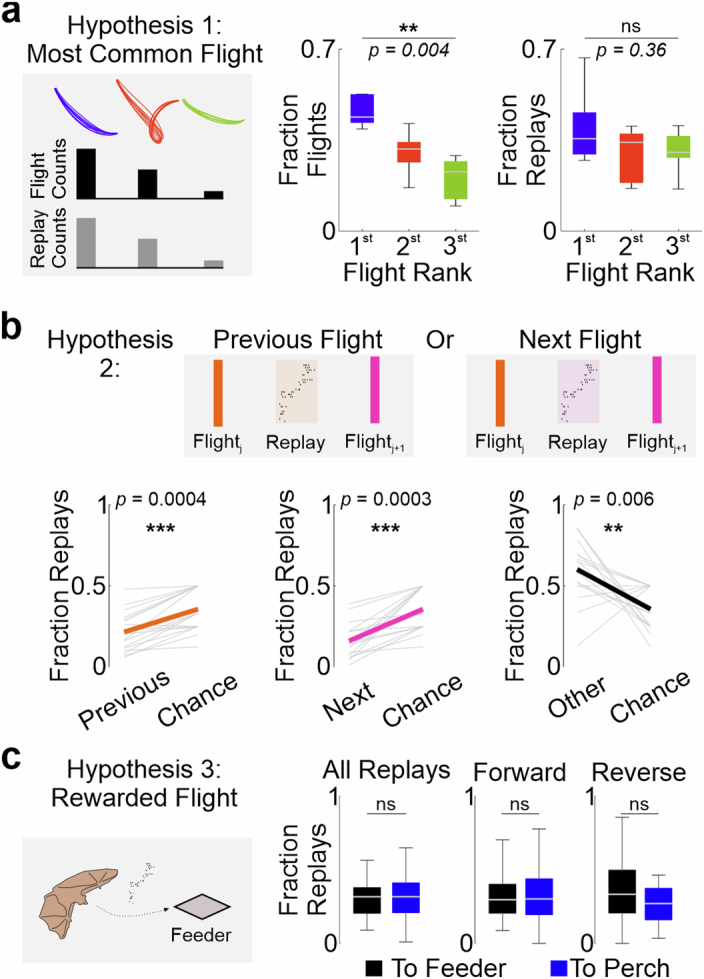
Simple models do not explain the relationship between replay content and behaviour. **a**, Most common flight hypothesis. Left: schematic illustrating the hypothesis that replays are more likely for the most frequently taken flight paths (e.g., blue path). Middle: fraction of flights belonging to the 1st, 2nd and 3rd most common trajectories, ranked by frequency within each session. Right: corresponding fraction of replays for each trajectory rank. Box plots show median (centre line), 25th–75th percentiles (box) and minimum and maximum (whiskers) across sessions (*n* = 9 sessions from 3 bats in which at least 3 different trajectory types were each replayed at least 15 times; Wilcoxon signed-rank test between 1st and 3rd ranks). Note that replay fraction does not scale with flight frequency. **b**, Top: schematic illustrating the hypothesis that replays reflect the immediately preceding (orange) or following (purple) flight. Bottom: fraction of replays classified as immediate previous (left), immediate next (centre), or other (right), compared with chance level (*n* = 17 sessions from 5 bats; Wilcoxon signed-rank test, Methods). Chance level for each session is calculated as one over the number of different flight trajectories in that session that could be replayed. Thin lines depict single sessions; thick lines show the averages. **c**, Rewarded flight hypothesis. Left: schematic illustrating the hypothesis that replays prioritize flights to rewarded locations (feeders). Right: fraction of replays corresponding to flights ending at feeders (black) or perches (blue), for all, forward, and reverse replays. Replay fractions are normalized by the number of feeder- or perch-bound flight types present in each session, so values do not sum to 1. Box plots as in **a** (*n* = 23 sessions from 6 bats; Wilcoxon signed-rank test). No significant difference was observed between replays for reward- and perch-directed flights. *P* values are two-sided. ***P < 0.001; **P < 0.01; *P < 0.05; ns: *P* > 0.05. Illustration in **c** adapted from ref. ^14^, Springer Nature Limited, under a Creative Commons licence CC BY 4.0.

During natural foraging, an animal’s behaviour can be segmented across multiple spatio-temporal dimensions, including the spatial features of movement patterns, resting locations and behavioural states (for example, movement versus rest). We made use of these inherent features of spontaneous foraging behaviour alongside the known structured spatial patterns of bats^13^ to investigate replay dynamics across space and time. First, within each session, bats repeatedly executed distinct and self-selected, yet highly structured flight trajectories^13^ (1–8 types, mean: 4 types per session, *n* = 23 sessions from 6 bats; Fig. 2a). Different ensembles of place cells participated in the representation of different trajectories (Extended Data Fig. 4), allowing us to uniquely assign a trajectory for every replay event (Fig. 2b). Next, we observed that bats naturally alternated between flight bouts and resting periods, each often lasting several minutes (Fig. 1b), and that replays tended to be more frequent during the latter (Fig. 2c). This allowed us to investigate the spatio-temporal nature of replay events and specifically ask whether replay occurred at times and locations that were proximal or distal to the spatial experience. We found that the replay rate was significantly higher during extended rest epochs than it was in time periods right before or after a flight (0.59 ± 0.06 replays per min during rest versus 0.39 ± 0.04 replays per min around flight times (within 30 s of a flight), *P* = 9.7 × 10^−8^, Wilcoxon signed-rank test; 19 sessions, 5 bats; Fig. 2d). Examining the relationship between the bat’s location during replay events and the start location of replayed trajectories (examples in Fig. 2e), we found that a substantial percentage of both forward and reverse replays occurred when the bat was in a remote location relative to the replayed trajectory (median fraction of remote forward replays: 0.69, *P* = 0.0037, Wilcoxon signed-rank test against 0.5; reverse replays: 0.69, *P* = 0.0029, Wilcoxon signed-rank test against 0.5; 2,155 forward replays, 1,620 reverse replays; 19 sessions from 5 bats; Fig. 2e, right). The nonlocal nature of replays during spontaneous foraging was even more pronounced when we combined both spatial and temporal proximity constraints, with only a small fraction of forward replays happening when the bat was spatio-temporally proximal to flight take-off (223 out of 2,050 forward replays (11%); Fig. 2f, left) or reverse replays when it was close to landing (150 out of 837 reverse replays (18%); Fig. 2f, right). Furthermore, analyses of the relationship between replay and ongoing spatial behaviour revealed that replay was not a simple recapitulation of the most frequent, recent or rewarded spatial experiences (Extended Data Fig. 5). Together, these findings suggest that during naturalistic foraging, most replay events are spatio-temporally dissociated from the replayed spatial experience.

**Extended Data Fig. 6:**
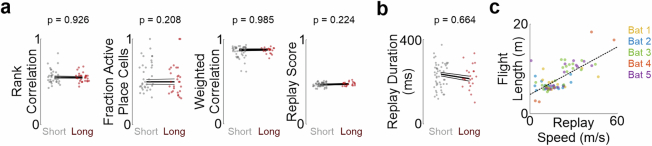
Replay features for flights of different lengths and durations. **a**, Comparison of different replay metrics (Methods) between short and long flights (*P* values are from Wilcoxon rank-sum test, *P* values are two-sided, *n* = 37 short versus 28 long flights for rank correlation and fraction active cells, obtained from spike-sequence analysis; *n* = 51 short versus 22 long flights for the remaining scores, obtained from decoding analysis). Here and in **b**, thick line represents median; thin lines represent s.e.m. **b**, Comparison of replay duration calculated from decoded replays between short (*n* = 51 trajectories) and long (n = 22 trajectories) flights. *P* values are two-sided. **c**, Flight length versus replay speed calculated from decoded replays. Black dotted line denotes the line of best fit. Note the linear increase in replay speed as flight length gets longer. Each dot represents the average replay duration for a single flight trajectory, colour-coded by the identity of the bat, *n* = 78 flight trajectories.

**Extended Data Fig. 7:**
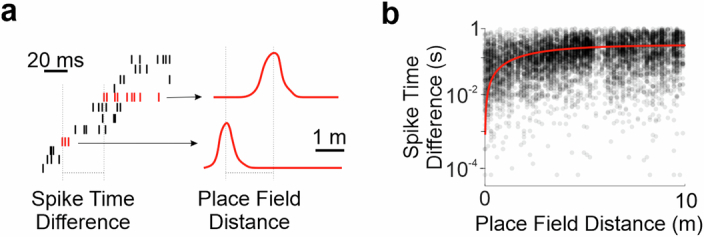
Distance between place fields versus timing between spikes during replay. **a**, Schematic showing the interval between first spikes and the distance between place fields for a specific neuron pair participating in a replay event. **b**, Interval between first spikes versus distance between place fields for all the place-cell pairs that participated in replay events fulfilling analysis criteria (8,328 valid pairs from 1,073 replays; Methods). Note that at short distances the time between first spikes increases with the distance between place fields, but this increase plateaus at distances of a few metres, in line with the approximate constancy of replay duration across varying lengths.

**Extended Data Fig. 8:**
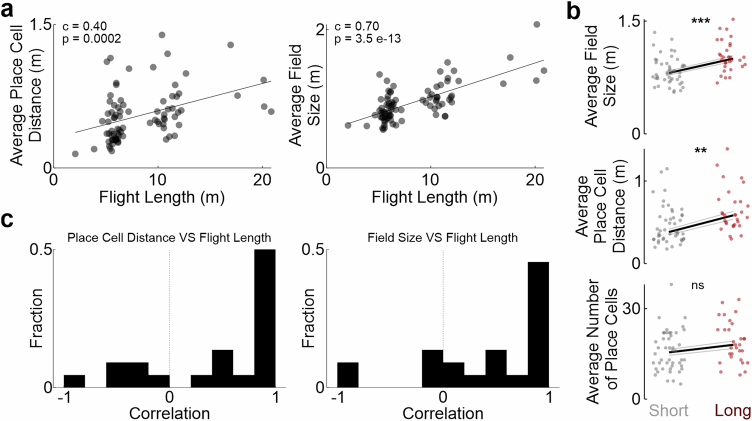
Place-field size and distance for flights of different lengths. **a**, Average distance between place cells (left) or average field size (right) versus flight length (Methods). Each marker represents the average value for place cells active on a flight cluster (*n* = 82 flight clusters from 23 sessions and 6 bats). Solid line represents linear fit. *c* and *p*: value and *P* value of Pearson’s correlation. *P* values are two-sided. **b**, Comparison between average field size (top), place-cell distance (middle) and number of recruited place cells (bottom) for short versus long flights (*n* = 46 short (3–7 m) versus *n* = 30 long (7–13 m) flights). Two-sided *P* values obtained from Wilcoxon rank-sum test: 7.9e-5, 0.001, 0.057 respectively. Thick line represents median, thin lines represent s.e.m. **c**, Distribution of single-session Pearson correlation values between average place-cell distance (left) or average place-field size (right) and flight trajectory length (*n* = 22 sessions from 6 bats with more than one analysable flight cluster). Note the highly skewed distributions, suggesting a positive correlation between field distance (or size) and trajectory length at the single-session level.

We also exploited the fact that, unlike in structured tasks, in which trajectories typically have a predetermined length (for example, on a fixed-length linear track), spontaneous foraging in three-dimensional (3D) open environments involves self-selected trajectories that can vary considerably in length. Indeed, the length of bat flight trajectories in the same experimental environment spanned nearly an order of magnitude (minimum, 2.7 m; maximum, 20.0 m; Fig. 2g), raising the question of whether replay duration scales with trajectory length. Although such scaling aligns with findings in rodents^5,20^, it presents a clear theoretical challenge in animals that naturally forage in large environments, which might necessitate replay events orders of magnitude longer than those reported in rodents previously^20^. We therefore examined the relationship between replay duration and flight length, focusing on the larger category of forward replays. Notably, average replay duration showed no appreciable increase with trajectory length (example replays in Fig. 2h; Pearson’s correlation *c* = 0.12, *P* = 0.33, *n* = 71 flight trajectories, 23 sessions, 6 bats). To quantify this phenomenon, we compared the average replay duration and speed (Methods) between two natural subdivisions of the flights (Fig. 2g): short flights (mean length 5.9 m; mean duration 2.15 s; grey distribution in Fig. 2i, top) and long flights (mean length 11.0 m; mean duration 3.85 s; red distribution in Fig. 2i, top) (length *P* = 7 × 10^−12^, duration *P* = 7 × 10^−12^, Wilcoxon rank-sum test). Although replay duration did not differ significantly between short and long flights (*P* = 0.796, Wilcoxon rank-sum test; Fig. 2i, bottom left), replay speed was significantly higher for long flights (*P* = 9.6 × 10^−5^, Wilcoxon rank-sum test; Fig. 2i, bottom right). Replay quality metrics did not differ significantly between the two groups (Extended Data Fig. 6a), ruling out differences in replay quality as an explanation for these results. Alternative measures of replay duration and speed, based on Bayesian decoding (Methods), yielded consistent findings (Extended Data Fig. 6b,c) and indicated a linear relationship between replay speed and trajectory length (Extended Data Fig. 6c). The constancy of replay duration was further supported by the asymptotic relationship between the timing differences in place-cell firing and the distances between the place fields of neuron pairs participating in the replay (Extended Data Fig. 7). Finally, we found an increase in both the average place-field size and the distance between place fields with increasing trajectory length (Extended Data Fig. 8), such that a similar number of cells spanned short and long flights, and their replays. Together, these findings indicate that replay dynamics are shaped mainly by internal mechanisms and vary only marginally by the duration or length of spatial behaviour.

## Cyclic sweeps of hippocampal representations

**Fig. 3:**
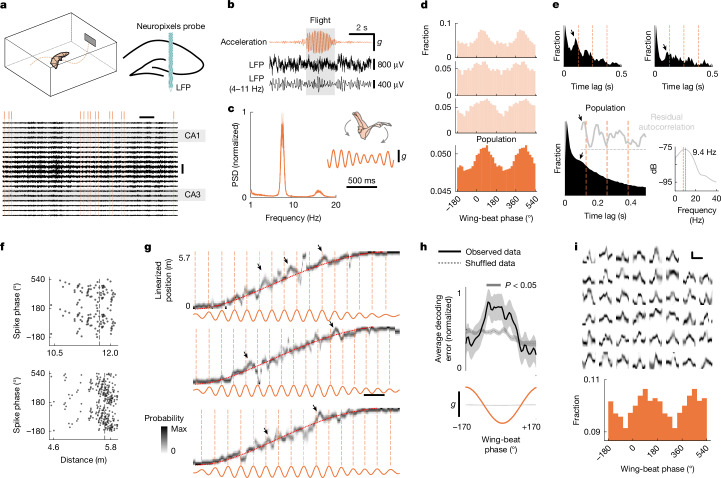
Oscillatory representation dynamics during flight and its relationship with the wing-beat cycle. **a**, Top, schematic of the aerial foraging experiment coupled with multi-site LFP recordings across the dorsal hippocampus. Bottom, representative epoch of the experiment, showing the LFP recorded across depths in the dorsal hippocampus (a subset of 20 channels, spanning the putative CA1–CA3 regions). Orange lines indicate flight times. Scale bars, 800 μV (vertical) and 1 min (horizontal). **b**, Top, example traces around flight time showing the bat’s absolute acceleration (orange) and average and filtered LFP (black middle and bottom traces) at the optimal probe contacts for putative theta (Methods). Note the absence of a prominent increase in theta oscillations during flight. **c**, Left, normalized average power spectral density of the raw accelerometer signal during flight (*n* = 22 sessions, 6 bats; Methods). Shaded area indicates s.e.m. Inset, example flight epoch showing the bat’s absolute acceleration. **d**, Example place cells and population average (bottom row; 326 neurons from 6 bats) showing phase locking to the wing-beat. Each histogram shows the fraction of spikes emitted at a certain wing-beat phase. Two cycles are shown for clarity. Bin size: 18.9°. Here, and in other panels, phase zero corresponds to the trough of the wing-beat (downstroke). **e**, Top, example auto-correlograms from phase-locked place cells. Each plot shows the fraction of spike intervals in a 10-ms time bin. Arrows indicate the prominent intrinsic oscillations at a frequency slightly higher than the wing-beat frequency (orange lines, fundamental and harmonics) for the same session. Bottom left, average auto-correlogram (black area) for all the phase-locked place cells (*n* = 326 neurons) and residual trace (grey trace) after subtracting the exponentially decaying component of the auto-correlogram (Methods). Arrows as in top panel. Bottom right inset, power spectral density (dB) of the residual autocorrelation trace, showing a prominent peak at 9.4 Hz (black vertical line), slightly higher than the wing-beat frequency (orange vertical line). **f**, Example place cells showing phase precession relative to the wing-beat oscillation. Each dot represents the phase (*y* axis) and distance along the flight (*x* axis) of a spike. Vertical lines represent the place-field centre. Spikes are represented twice for clarity, with a 360° phase shift. **g**, Decoded position during example flights. Grey colour map indicates the posterior probability (Methods) of linearized position along the flight. Red dashed lines indicate the actual position of the bat. Vertical dashed lines are the wing-beat cycles and orange trace shows the oscillatory accelerometer signal. Arrows indicate clear and large-amplitude decoded sweeps. Scale bar, 200 ms. **h**, Average decoding error (black trace) across all wing-beat cycles fulfilling inclusion criteria (5,141 cycles from 565 flights, 6 bats; Methods), aligned to the wing-beat accelerometer signal (orange trace). Dotted lines indicate the average decoding error obtained from shuffled data (Methods). Solid lines indicate mean; shaded areas are s.e.m. **i**, Top, example decoded probabilities for automatically detected sweeps (Methods). Bottom, wing-beat phase distribution for the central point of automatically detected sweeps (*n* = 1,367 sweeps, from 49 trajectories, 18 sessions, 6 bats). The central point of each sweep was determined using a template matching algorithm with a Gaussian-shaped template (Methods). Two cycles are shown for clarity. Bin size: 36°. Scale bars, 1 m (vertical) and 0.1 s (horizontal). Illustrations in **a** and **c** adapted from ref. ^14^, Springer Nature Limited, under a Creative Commons licence CC BY 4.0. Source Data

**Extended Data Fig. 9:**
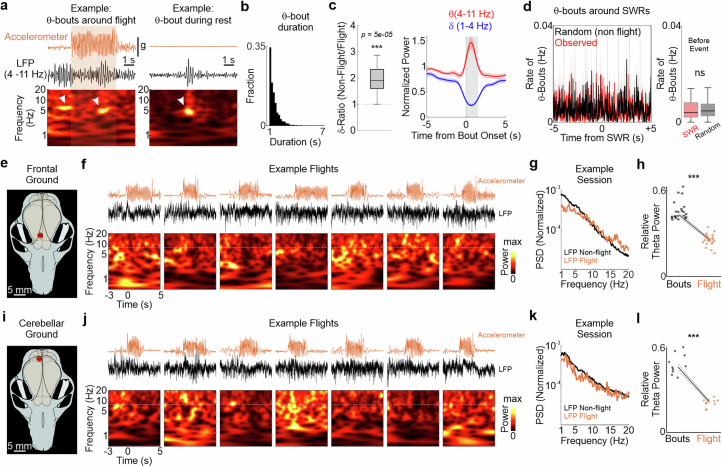
Hippocampal LFPs in bats lack continuous theta oscillations during flight, independent of reference site. **a**, Example theta bouts around and during flight (left), and during rest (right). For each example, absolute acceleration filtered between 7 and 9 Hz (top, orange trace), LFP trace (middle, black trace) and spectrogram (bottom) are shown. White arrows indicate theta bouts (Methods). **b**, Distribution of all theta bouts durations, including rest and flight time (1,512 bouts from 22 sessions and 6 bats). **c**, Left, box plot showing the ratio of delta power during non-flight versus flight. The box plot shows the maximum and minimum values (whiskers), median (centre line) and 25th to 75th percentiles (box limits) across sessions (*n* = 22 from 6 bats). *P* value is for a two-sided Wilcoxon signed-rank test against unit ratio. Right: time course of theta (red) and delta (blue) power around the onset of theta bouts, normalized to their average values during randomly chosen rest epochs. Shaded grey rectangle indicates theta bout duration (average ± s.e.m.: 1.53 ± 0.02 s). Shaded areas indicate s.e.m. **d**, Left: average rate of theta bouts around observed SWRs (red) and random epochs during non-flight (control, black). The average rate in each time bin (555 ms) is compared between observed data and control (two-sided Wilcoxon signed-rank test; *n* = 22 sessions from 6 bats). Difference is not significant in all epochs. Note the extremely low rate of theta bouts around SWRs. Right: box plot showing the average rate of theta bouts in the epochs preceding SWRs (from −5 to 0 s, red) or random events (same interval, black). *P* = 0.14 Wilcoxon signed-rank test, *n* = 22 sessions from 6 bats. **e**, Schematics diagram showing the reference ground screw (red) positioning above the frontal cortex. Transparency shows brain outline. **f**, Absolute acceleration (top), unfiltered LFP trace (middle) and spectrogram (bottom) around take-off for seven example flights, where LFP was referenced to a frontal ground. Note the absence of theta during flight (217 out of 2,128 flights with theta, 10%, *n* = 6 bats, 22 sessions; Methods). **g**, Power spectral density (PSD) of the LFP during epochs of non-flight (black trace) and flight (orange trace) for one example session where LFP was referenced to a frontal ground. Note the absence of prominent peaks in the theta frequency range (4–11 Hz). **h**, Power in the 4–11 Hz band, relative to power across the whole spectrum during theta bouts (bouts) versus during flight (Flight). *P* = 4e-5 two-sided Wilcoxon signed-rank test, *n* = 22 sessions from 6 bats. Thick line represents median; thin lines represent s.e.m. **i**–**k**, Same as in **e**–**g**, but for data collected with the reference ground screw (red) positioned above the cerebellum (two bats). Note, as in **f**,**g**, the absence of theta during flight (11 out of 344 flights with theta, 3%, *n* = 2 bats, 10 sessions; Methods). **l**, Same as **h**, but with LFP referenced to a cerebellar ground. *P* = 2e-4 Wilcoxon signed-rank test, n = 10 sessions from 2 bats.

Replay events occur predominantly during periods of immobility, but it is unclear how neural ensembles are organized during ongoing movement. Theta sequences^6,23^ are a key phenomenon thought to organize temporally compressed neural sequences during movement, and have been proposed to causally support the generation of replay events^7,8^. Because theta sequences are, by definition, thought to rely on theta oscillations^7^, it remains unclear how such a mechanism can generalize across species that differ markedly from rodents in theta oscillatory patterns. Bats present a challenge to such models, owing to the apparent absence of continuous theta oscillations in the bat hippocampus during movement^11,17,24^. However, so far, all studies of oscillatory LFP dynamics in bats been performed in either crawling or stationary individuals, leaving open the possibility that theta oscillations exist in the bat’s most natural movement state; that is, during flight^25^. To address this gap, we examined hippocampal LFP directly during flight, overcoming the challenges posed by flight-related artefacts that led previous studies to exclude such data^17,24^. The long shanks of Neuropixels probes, combined with the minimal incidence of flight-related artefacts, enabled us to densely sample high-quality LFPs from the hippocampus during aerial foraging (*n* = 8 bats, 32 sessions; Fig. 3a). Theta oscillations were mostly undetectable during flight (Fig. 3b), occurred in short bouts of increased theta-to-delta ratio mainly during rest and were temporally dissociated from SWRs (Extended Data Fig. 9a–d). Only a small fraction of flights had detectable theta oscillations (228 out of 2,472 (9%), *n* = 8 bats, 32 sessions; Methods), regardless of the location of the reference ground (frontal or cerebellar ground, Extended Data Fig. 9e–g,i–k). Furthermore, under both referencing configurations, the LFP theta power during flight was significantly lower than it was during spontaneous theta bouts (frontal ground *P* = 4 × 10^−5^, cerebellar ground *P* = 2 × 10^−4^, Wilcoxon signed-rank test; Extended Data Fig. 9h,l), which occurred mainly during rest (fraction of theta events detected during rest, frontal ground: 73 ± 4% (mean ± s.e.m.), 22 sessions from 6 bats; cerebellar ground: 93 ± 2% (mean ± s.e.m.), 10 sessions from 2 bats). These findings reveal that even during flight, continuous theta oscillations are absent in the bat hippocampus.

**Extended Data Fig. 10:**
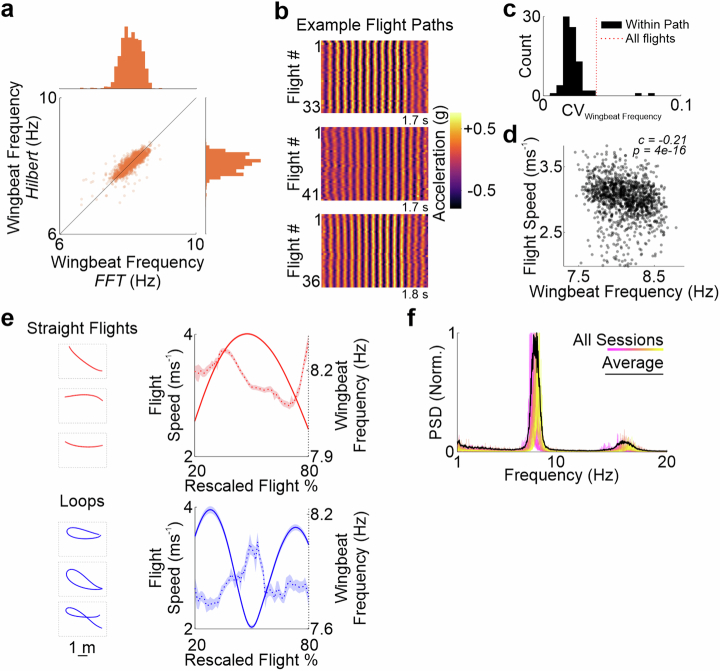
Limited wing-beat frequency variability across flights. **a**, Scatter plot and histogram for the wing-beat frequency calculated from the FFT of the absolute acceleration during a flight (vertical axis) or as the average during flight of the time-varying frequency obtained from the Hilbert transform (Methods). Each dot is one flight (*n* = 1,442 flights from 6 bats). Note the high correlation between the two measures and their narrow distributions. **b**, Heat maps representing the accelerometer signal (filtered between 7 Hz and 9 Hz) during repeated executions of the same flight in a path (three example paths from different bats). Number below the heat map indicates duration of the shown flight interval. **c**, Distribution of the coefficient of variation (CV) for the wing-beat frequency across flights belonging to the same path (*n* = 80 flight paths from 6 bats). The vertical line represents the coefficient of variation of the wing-beat frequency across all flights (*n* = 1,442 flights from 6 bats). Note that most of the coefficients of variation of wing-beat frequency within a path were significantly smaller than the coefficient of variation across all flights (97.5%, *P* = 0.025). **d**, Scatter plot for the average speed versus average wing-beat frequency during flight (each dot is a flight, *n* = 1,442 flights from 6 bats). To improve visibility, only data within 5th–99th percentiles are shown. *c* and *p*: value and two-sided *P* value of Spearman’s correlation. **e**, Example trajectories (left insets), average speed profile (solid line) and average wing-beat frequency (dotted line) for straight flights (*n* = 1,057, top) versus loops (*n* = 385, bottom; Methods). Shaded areas represent s.e.m. Average profiles are calculated by rescaling the speed (or wing-beat frequency) between 0 (take-off) and 100 (landing) and then averaging the obtained traces across flights. Flight tails are excluded to avoid edge effects. **f**, Unfiltered power spectral densities of the raw accelerometer signal during flight for all sessions (*n* = 22, 6 bats; coloured lines, scaled between 0 and 1). Black line shows the average.

We next considered whether alternative mechanisms might interact with ensemble activity on the fast timescales that are characteristic of theta sequences and replays. By analysing accelerometer data during foraging sessions (Methods), we observed another oscillatory pattern accompanied by a prominent rhythm at about 8 Hz that was associated with the behaviour of the bat during flight: the wing-beat cycle^17,26^ (Fig. 3c), the frequency of which was very consistent across individuals and flight trajectories (Extended Data Fig. 10), akin to the high consistency of stepping in rats^27^. Studies have suggested that behavioural movement patterns, such as stepping^27^, head oscillations^28^ and whisking^29^ in rats or gaze movements in primates^30,31^, can influence neural representations in the hippocampus. We therefore investigated the relationship between ensemble activity during flight and the motor (wing-beat) aspects of the bat’s behaviour. Notably we observed that many place cells exhibited phase locking to the wing-beat cycle (326 out of 875 neurons (37%) from 6 bats; example neurons in Fig. 3d; Methods), with most neurons activating around the ascending phase of the wing-beat (maximum mean firing at 71° (95% confidence intervals: 61°–80°); *n* = 326 neurons from 6 bats; Fig. 3d, bottom, corrected for uneven phase distribution; Methods), suggesting a population-level phase-locking mechanism. Notably, phase-locked neurons were present even when using more stringent criteria, based on Rayleigh statistics (average: 15%, *n* = 22 sessions from 6 bats, *P* < 0.05 from shuffling analysis, resultant vector length: 0.13 ± 0.01 (mean ± s.e.m.), Rayleigh statistics: 7.6 ± 2.2, spike count: 666 ± 63; Methods), confirming the robustness of the phenomenon. Furthermore, both the single-neuron and the average autocorrelation function of the phase-locked neurons revealed a slightly faster intrinsic oscillatory rhythm (dominant frequency: 9.4 Hz, *n* = 326 neurons from 6 bats; Methods and Fig. 3e), consistent with resonance or adaptation dynamics. Together, these findings predict two phenomena that were previously linked to oscillatory patterns in the LFP: behavioural phase precession, in which spike timing relative to the wing-beat advances as the bat traverses the place field; and fast oscillatory sweeps, analogous to theta sequences, but coordinated with the wing-beat cycle. We therefore sought to investigate the existence of these phenomena.

**Extended Data Fig. 11:**
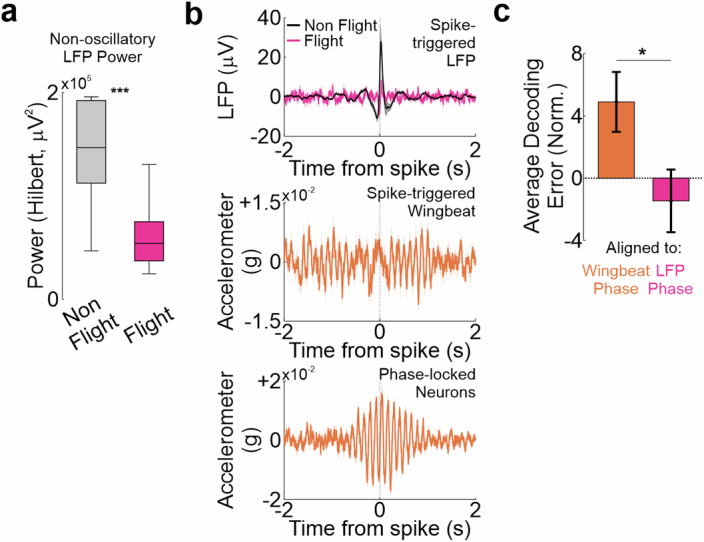
Comparison between non-oscillatory LFP and wing-beat phase locking of hippocampal neurons. **a**, Mean power of the non-oscillatory LFP (Methods) during non-flight versus flight epochs. The box plot shows the maximum and minimum values (whiskers), median (centre line) and the 25th to 75th percentiles (box limits) across sessions (two-sided *p* = 4e-5, n = 22 sessions from 6 bats, Wilcoxon signed-rank test). **b**, Top, spike-triggered LFP, computed as the mean of the single-session spike-triggered LFP traces (*n* = 22 sessions from 6 bats) for all the spikes emitted during non-flight (black) versus flight (purple). Shaded areas represent s.e.m. Middle: Same as above, but for the spike-triggered wing-beat. Bottom: Same as above but only including spikes from phase-locked neurons (*n* = 22 sessions from 6 bats; Methods). **c**, Average decoding error (Methods) aligned to either the wing-beat phase (orange bar) or the non-oscillatory LFP phase (purple bar) and normalized to a shuffled distribution (two-sided *P* = 0.044 Wilcoxon rank-sum test, *n* = 4,546 wing-beat cycles versus 4,393 LFP cycles from 643 flights, 6 bats). Bars indicate the mean, error bars are s.e.m.

**Extended Data Fig. 12:**
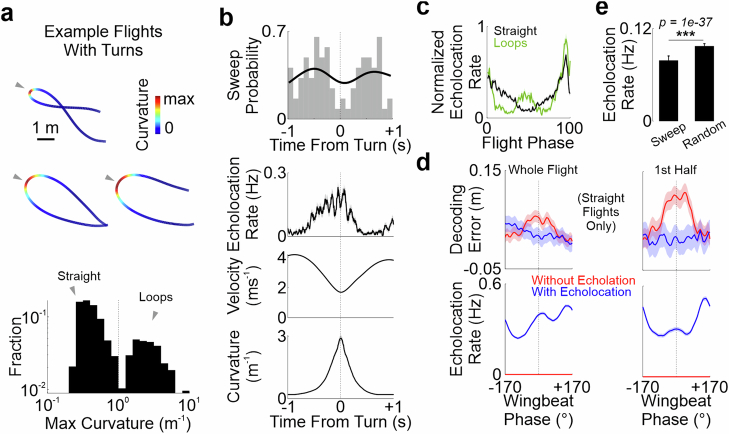
Fast representational sequences around turns and echolocation. **a**, Top: Example flights with turns, shown in top view. The colour at each position indicates the local 3D curvature. Grey triangles mark the turn point, defined as the location of maximum curvature (Methods). Bottom: Distribution of maximum curvature across flights (*n* = 1,354 flights from 22 sessions, 6 bats). The vertical line denotes the threshold value of 1 m^−1^ used to distinguish loops from straight flights. **b**, Top: Distribution of representational sequence (sweep) occurrence within a [−1, 1] s window around detected turns (*n* = 122 automatically detected sweeps, 18 flights with turns, 6 sessions, 5 bats; median turn time from start/stop of flight: 1.9 s/1.7 s). Black line shows the kernel density estimation. Bottom: Average echolocation rate, velocity and curvature aligned to the turn point (*n* = 274 flights with turns from 10 sessions, 4 bats). Thick lines represent the mean; shaded areas denote s.e.m. **c**, Average echolocation rate across normalized flight phase (%; 0 = take-off, 100 = landing) for straight flights versus loops (*n* = 880 straight flights from 16 sessions, 4 bats; *n* = 274 flights with turns from 10 sessions, 4 bats). Each echolocation trace was normalized to its maximum value before averaging. Note the transient increase in echolocation rate near the midpoint of loops. Thick lines indicate mean; shaded areas denote s.e.m. **d**, Average decoding error (top) and echolocation rate (bottom) across wing-beat cycles from straight flights (*n* = 591 straight flights from 15 sessions, 4 bats). Traces are aligned to the phase of the wing-beat and split into cycles with (blue, *n* = 3,451 cycles for whole flight, *n* = 1,712 for 1st half) versus without (red, *n* = 6,184 cycles for whole flight, *n* = 3,166 for 1st half) echolocation. Plots on the right include only wing-beat cycles from the first half of flights. Solid lines indicate mean; shaded areas indicate s.e.m. Note the reduction in decoding error during cycles with echolocation. **e**, Average echolocation rate in a [−40:40] ms window around observed sweeps versus randomly selected time points (two-sided Wilcoxon sign rank test, *n* = 1,214 sweeps, 4 bats; Methods). Bars indicate the mean, error bars are s.e.m.

**Extended Data Fig. 13:**
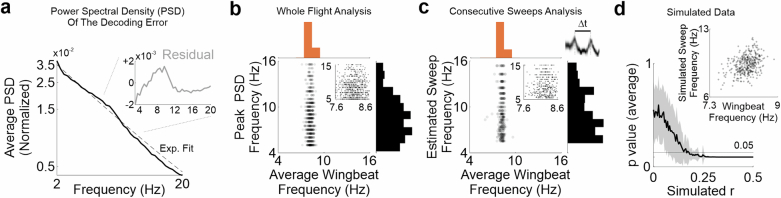
Relationship between the frequency of wing-beat and of internal representations. **a**, Average power spectral density (PSD, normalized to unit area) for the decoding error during flight (*n* = 659 flights from 16 sessions and 6 bats). Dashed line shows fit with exponential decay. The inset shows the residual after subtracting the exponential decay. Note the peak around 8 Hz. **b**, Scatter plot and histograms for the peak of the residual PSD of the decoding error (after subtracting the exponential decay) versus average wing-beat frequency for single flights (dots, *n* = 659 flights from 16 sessions and 6 bats; Methods). Inset shows the same data at higher magnification. **c**, Same as **b**, but for the estimated sweep frequency for consecutive sweeps (example in the topmost inset, calculated as the inverse of their time interval, *n* = 360 from 15 sessions, 5 bats) versus average wing-beat frequency calculated from the time average of the instantaneous wing-beat frequency on the same time interval between sweeps (Methods). Inset shows the same data at higher magnification. **d**, Average *P* value (calculated over 30 repetitions, shaded area indicates s.d.) for the Spearman correlation between simulated sweep frequency versus empirical wing-beat frequency, plotted against imposed correlation values. Simulated sweep frequency (*f*) was generated from the empirical wing-beat frequency values (*x*) as *f* = *r·*zscore(*x*) + *√*(1 − *r*2) *·z* *+* mean(x), where *z* ≈ *N*(0,1). Inset shows data for one simulated sample at *r* = 0.5. Simulations suggest that even under idealized conditions, detecting weak correlations (e.g., *r* ≈ 0.2, similar to the value observed between stepping and theta in rats^27^) could be challenging, given the little wing-beat frequency variability.

Behavioural phase precession was prominent in a substantial fraction of phase-locked place cells (147 out of 326 neurons (45%) from 6 bats; examples in Fig. 3f). Furthermore, decoding the bat’s position from ensemble activity during flight (Methods) revealed clear representational sweeps (Fig. 3g), in which the decoded position moved ahead of the bat’s current position cyclically before returning to it, reminiscent of theta sequences in rodents^6,27^. We noticed that large amplitude and clearly defined sweeps (arrows in Fig. 3g) often occurred at a consistent phase of the wing-beat, as predicted by our hypothesis. We used two complementary approaches to examine the phase locking of sweeps to the wing-beat cycle. First, we averaged the decoding error (decoded position minus actual position) across all cycles of the wing-beat (5,141 cycles from 565 flights, 6 bats; Methods) and compared it with a shuffled distribution, obtained by randomly shifting the wing-beat phase at each cycle by up to ±60 ms (Methods). We found that the average decoding error was significantly larger than the shuffled distribution around the trough of the wing-beat cycle (*P* < 0.05; Fig. 3h). Applying the same alignment procedure but relative to cycles of the non-oscillatory LFP phase (Methods) resulted in a significantly smaller average decoding error (Extended Data Fig. 11), suggesting that non-oscillatory phase-locking mechanisms^24^ may be more relevant in the absence of prominent behavioural rhythms, such as during immobility or crawling in bats. This finding was consistent with the significant decrease in non-oscillatory LFP power observed during flight, as well as with the absence of the spike-triggered LFP relationship relative to non-flight (Extended Data Fig. 11a,b and Methods). Next, we used an automatic detection algorithm (Methods) to extract all decoded events with a stereotypical sweep profile (Fig. 3i, top) and quantified their phase relative to the wing-beat (*n* = 1,367 sweeps, from 49 trajectories, 18 sessions, 6 bats). The resulting distribution showed a clear peak in the ascending phase of the wing-beat (peak at a mean of 116° (95% confidence intervals: 63°–131°); Fig. 3i, bottom). Intriguingly, we found that the expression of sweeps was related to ongoing flight behaviour, with sweep occurrence decreasing during turns (Extended Data Fig. 12a,b and Methods). Because flight turning events were typically accompanied by an increased echolocation rate (Extended Data Fig. 12b,c), we investigated whether active sensing, rather than locomotion per se, might affect sweep expression by restricting our analysis to flights lacking turns (that is, straight flights). In line with this hypothesis, we found that the stereotyped decoding error profile observed in relation to wing-beat cycles was disrupted during echolocation, as compared with wing-beat cycles without echolocation (Extended Data Fig. 12d). Consistent with this observation, we found that automatically detected sweeps (Methods) were more likely to occur during periods of significantly lower echolocation rate than at randomly selected time points during movement (Extended Data Fig. 12e). This suggests that active sensory sampling, during which bats are at a state of heightened attention^32^, transiently interferes with the expression of internal representations, which, in turn, can be dynamically regulated on a cycle-by-cycle basis. Finally, although both wing-beat and sweep dynamics showed approximately 8-Hz rhythms, their frequencies did not covary across or within flights—an effect that might reflect the limited variability in wing-beat frequency, potentially obscuring the detection of subtle correlations (Extended Data Fig. 13).

Together, these results confirm the existence of fast and cyclic representational sequences that might be related to a behaviourally relevant sensorimotor rhythm, rather than a neural oscillatory rhythm (that is, theta oscillations). These findings further broaden the applicability of such ensemble computations to species that do not show continuous theta oscillations, but exhibit clear behavioural rhythms^30,33^—including non-human primates^12^ and humans^34^.

## Discussion

By recording large-scale ensemble activity and LFPs from the hippocampus of freely behaving and flying bats, we challenge established models of replay and oscillatory representation dynamics, and propose a new framework, in which behaviourally relevant sensorimotor rhythms interact with hippocampal ensemble activity in a temporally structured manner. Although replay-like activity (reactivation) has been observed across species, including in humans^18,35^, the specific form of temporally compressed, sequential hippocampal replay—widely considered to be foundational to models of memory consolidation, planning and cognitive map updating^36^—has so far been robustly demonstrated only in rodents. Thus, the generality of these phenomena across species that differ from rodents in their behaviour and neurophysiology has remained largely unknown. Here, we leveraged the natural spatial behaviour of Egyptian fruit bats to reveal temporally compressed, sequential hippocampal ensemble phenomena—replay and representational sweeps—that deviated considerably from predictions based on widely accepted models. We found that although basic features of ensemble dynamics, such as the existence of both forward and reverse replays and their correspondence with SWRs, confirm previous findings in rodents, fundamental aspects, such as the relationship between replay duration and trajectory length, or the cyclic organization of neural representations in the absence of rhythmic LFP, suggest that other underlying mechanisms exist.

Our finding that replay duration is nearly constant across spatial scales that span an order of magnitude is in clear contrast with experimental results in rodents, in which replay duration has been reported to scale linearly with the length of spatial behaviour^5,20^. However, studies showing that replay features are more dynamic than previously thought^37–39^ align with our results, suggesting that replay is more than a linear chaining of representations. These findings highlight the importance of performing studies under ethologically relevant, unconstrained conditions when investigating the neural mechanisms that underlie spatial behaviours^40,41^. The spatial scales investigated here reflect the natural foraging behaviour of Egyptian fruit bats at local feeding sites^42^. Our findings suggest that the approximately constant replay duration we observed represents an elemental unit of information processing. The same mechanism might also extend to replays of longer trajectories, such as those occurring during large-scale commutes in this species—which can occur on the scale of dozens of kilometres^42,43^—and might be segmented into these fundamental chunks, providing a mechanism for efficient coding during large-scale navigation. Future studies investigating navigation across larger spatial scales and the multi-scale nature of place fields^44^ could test this hypothesis, offering further insights into the hierarchical organization of spatial memory.

By leveraging recording technologies that enable monitoring of hippocampal LFPs during all states of motion in bats, we observed a lack of sustained theta oscillations during flight. This finding challenges existing models linking replay and cyclic neural sequences during movement to theta oscillations^7,8,10^. Although non-oscillatory dynamics in bats^24^ have been proposed to explain phase locking and phase precession at the single-cell level, these were based solely on LFP data from crawling or stationary bats, and, notably, did not address the organization of ensemble activity that we observed. By contrast, our data provide evidence that non-oscillatory LFP dynamics are substantially attenuated during flight, and that the spike–LFP relationships previously reported^24^ are effectively absent. Instead, the wing-beat rhythm provides a more consistent temporal reference, with stronger phase relationships to internal representations. These results suggest that in bats, behavioural rhythms interact strongly with hippocampal activity during flight, on timescales that are conducive to synaptic plasticity^10^, whereas during rest, in the absence of such rhythms, internally generated LFP dynamics may be more prominent^45^. The mechanistic and circuit-level basis of how the wing-beat rhythm can influence neural activity without a prominent LFP signature has yet to be determined. Potential mechanisms include cyclic neuromodulatory signals or oscillatory synaptic inputs from cortical regions that govern the bat’s sensorimotor behaviour (for example, the motor cortex). This notion also aligns with findings in rodents, in which locomotion can dynamically entrain hippocampal representations^27^, potentially also through mid-brain nuclei^46^. Furthermore, continuous motor behaviours rely on ongoing sensory feedback (for example, stepping in rodents^47^ or echolocation in bats^48^), and both sensory and motor components are ideally suited to modulate neural activity. We observed that an increased echolocation rate corresponded to a reduced expression of coherent internal sequences, possibly through a rapid interplay between internal and external processes modulated by attentional demands. Future studies in spatial environments of varying complexity (for example, using obstacles) will be important to further examine how wing motion and active sensing (echolocation) interface with ensemble dynamics to support navigation. Advancing technologies that enable simultaneous recordings of larger neural populations, spanning several brain regions, will be crucial to unravel how areas such as the motor cortex or entorhinal cortex^49^ contribute to hippocampal ensemble activity in bats and other animals. At the same time, the relationship between motor rhythms and hippocampal activity might be shaped by the distinct behavioural repertoires of animal species. Indeed, even within a single species, different types of hippocampal oscillations can emerge under different conditions^50^, and a wide range of species-specific motor rhythms—such as whisking, sniffing or saccades—can modulate hippocampal dynamics^27–30^. These complexities demonstrate the need for cross-species comparisons, and highlight the power of comparative approaches in uncovering both conserved and specialized mechanisms of spatial navigation and memory^40,41^.

## Methods

### Bats

**Extended Data Fig. 14:**
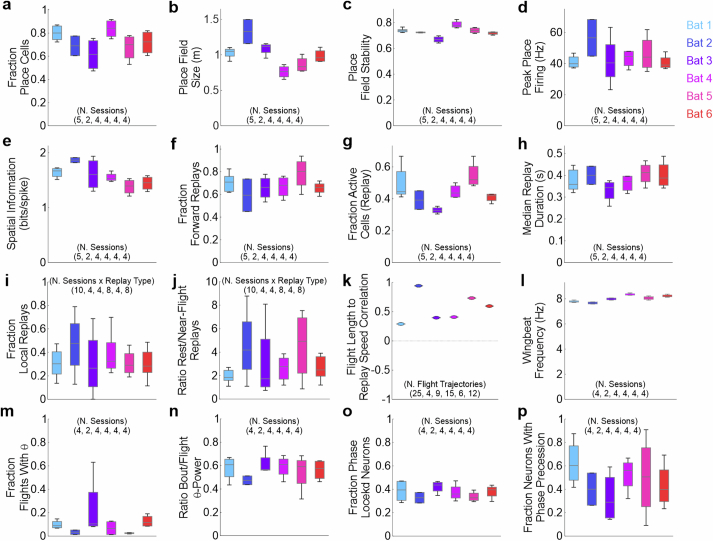
Statistics for individual bats for spatial coding, replay and oscillatory dynamics variables. **a**–**p**, Box plots showing individual statistics for several variables. Each box plot, excluding **k** (see below), shows the maximum and minimum values (whiskers), median (centre line), and 25th to 75th percentiles (box limits) across sessions (or session × replay type: forward or reverse; numbers in parentheses). The plot in **k** shows, for each bat, the Pearson correlation between flight trajectory length and the median replay speed for that flight (number of flight trajectories used to calculate the correlation in parentheses). Note the strongly skewed distribution of correlations toward positive values across bats, suggesting that replay speed increases with trajectory length. Bats 2 and 3 were flown in the outdoor enclosure; the remaining bats were flown in the indoor flight room (see Methods).

Experiments involved a total of eight adult male Egyptian fruit bats (*Rousettus aegyptiacus*; body weight around 151–171 g). Six bats were used for the analysis of single units and LFP (*n* = 5 bats implanted with 3 Neuropixels probes, *n* = 1 bat implanted with 2 Neuropixels probes). We collected 23 sessions (3–4 sessions per bat), during which neural activity was recorded during rewarded aerial foraging. Individual bat statistics for several measures related to spatial coding and ensemble phenomena are reported in Extended Data Fig. 14. Two additional bats were used for examining LFPs referenced to a cerebellar ground screw (see ‘LFP recordings with cerebellar ground’). All bats were housed in a humidity- and temperature-controlled room. Implanted bats were single housed after implant surgery. Lights in the housing room were maintained on a 12-h–12-h reverse light cycle (lights off–lights on; 07:00–19:00). All experiments were performed at the same time of day during their awake hours (dark cycle). All experimental procedures were approved by the Institutional Animal Care and Use Committee at the University of California, Berkeley.

### Aerial foraging

Foraging experiments took place in an indoor flight room (*n* = 4 bats, 5.6 m × 5.2 m × 2.5 m) or an outdoor flight enclosure (*n* = 2 bats, 10.1 m × 4.1 m × 2.8 m). All bats were mildly food-restricted (>85% of their baseline weight) during training and recording sessions. Training (three to nine days before neural recordings) consisted of 60–120-min daily sessions in which the bats could spontaneously obtain feeder-dispensed puréed reward by landing on designated platforms. No humans were inside any of the flight enclosures during experiments to avoid human-induced experimental confounding^51^, unless otherwise stated. Recording sessions lasted between 70 min and 115 min.

The indoor flight room was an acoustically, electrically and radio-frequency shielded room with high-precision lighting control. The flight room ceiling and walls were covered with acoustic foam to minimize acoustic reverberation and dampen noise from adjacent rooms. An additional layer of acoustically absorbing black felt was placed around the walls and the floor to protect the acoustic foam from being damaged by the bats. The 3D spatial position of the bats was tracked at millimetre resolution using 16 motion-capture cameras^13,52^ (Raptor-12HS, Motion Analysis). Each camera tracked three reflective markers, attached to the neural recording headstage on the head of the bat, at a frame rate of 120 Hz. The 3D position of the marker-set centroid was acquired using commercially available software (Cortex-64; Motion Analysis). Two automated feeders placed on the wall at one end of the room dispensed a puréed fruit reward. Reward was triggered when a bat landed on the feeding platform and interrupted an infrared beam break sensor mounted in front on the reward port. Feeders were all independently controlled by an Arduino (Uno Rev3) and Adafruit Motorshield (1438; Adafruit) interfaced with a computer outside the experimental room. Reward probability (0.2–0.8) and amount (0.1–0.3 ml) were adjusted by the experimenter to fine-tune the bat’s behaviour. On a subset of sessions (*n* = 6 sessions from 3 bats), barriers were added in the room or lights were turned off to encourage the execution of new flight trajectories. All remaining sessions were performed under uniform illumination (luminance level 5 lux) and without barriers.

Two bats participated in foraging experiments in an outdoor flight enclosure, following a similar procedure. One computer-controlled feeder was remotely triggered by the experimenter after the bat landed on an elevated platform. The 3D spatial position of each bat was recorded using a modified version of a commercial real-time location system^14^ (RTLS; Ciholas). In brief, the system was composed of a mobile tag (DWTAG100), mounted on the neural recording headstage, that was localized at a 100-Hz sampling rate by 13 static anchors (DWETH101), communicating through ultra-wideband pulses. One additional anchor (custom DWETH101) was used to record an external synchronization signal. Tags were made of a lightweight (around 2.9 g) transceiver and a LiPo battery. The system communicated with a computer located outside the experimental enclosure through UDP protocol. The system was configured and operated through a web-based user interface running on Ubuntu 18.04 Bionic. Data were recorded and saved using custom-written scripts in Python. For all experiments, periodic clock pulses generated by a Master-9 device (A.M.P.I.) were used to create a timing signature that served as a common frame of reference for all the recording systems (tracking, neural recordings and audio; see below). Accelerometer data were acquired at 30 kHz by the neural recording headstage and downsampled to 500 Hz for analysis.

### Surgery

Probe implants were performed in two stages, separated by 7–10 days: (i) implantation of the training cone; and (ii) insertion of Neuropixels 1.0 probes.

#### Implantation of training cone

The bat was anaesthetized using an injectable cocktail of ketamine, dexmedetomidine (reversed by atipamezole) and midazolam (reversed by flumazenil). It was then placed on a stereotaxic apparatus (Model 942; Kopf) and provided with a continuous supply of oxygen. Anaesthesia was maintained by injections of a cocktail of dexmedetomidine, midazolam, and fentanyl (about once per hour). Anaesthesia depth was continuously monitored by toe-pinch reaction test and by measurements of the bat’s breathing rate. Body temperature was measured with a rectal temperature probe and maintained at approximately 35 °C using a regulated heating pad. After the correct anaesthetic depth was reached, the skull was exposed, and the surrounding skin and tissue were retracted. The exposed skull was cleaned of any residual connective tissue and scored to improve cement adhesion. A ground screw, which consists of a bone screw (19010-00; FST) with two or three stainless-steel wires (203.2 μm coated; A-M Systems) soldered to the screw head, was inserted into the frontal plate of the skull and served as the ground for each Neuropixels probe (one wire per probe). In two bats, used for LFP analyses only, the ground screw was inserted posterior to the sinus, above the cerebellum (see ‘LFP recordings with cerebellar ground’). Four shorter bone screws (M1.59 mm stainless steel) were placed to further strengthen the attachment of the implant to the skull. A circular 1-mm craniotomy was made for each probe insertion point, up to three craniotomies per bat (two symmetrical bilateral sites for two probes, plus one additional site in the right hemisphere in the case of three probes, all above dorsal CA1 at approximately 6.3 mm anterior to the transverse sinus that runs between the posterior part of the cortex and the cerebellum and 3.2 mm lateral to the midline). The craniotomy was then sealed with a biocompatible elastomer (Kwik-Sil; World Precision Instruments) to protect the brain surface until probe insertion. The skull and bone screws were covered with a thin layer of bone cement (C&B Metabond; Parkell). A custom 3D-printed cone was positioned and cemented using dental acrylic at three points of contact (to facilitate cone removal before probe insertion), and the remaining gaps sealed with biocompatible elastomer (Kwik-Sil; World Precision Instruments). The cone was closed with a custom 3D-printed cap. At the end of the surgery, reversal agents were injected to counteract the dexmedetomidine and midazolam, and after the bat had fully awoken from the anaesthesia, an oral analgesic (Metacam; Boehringer Ingelheim), was administered. Analgesics (three days) and antibiotics (seven days) were given daily until complete recovery. Behavioural training was resumed after the bat was allowed to fully recover from surgery for three days. During training, the weight of the implant was gradually increased over seven to ten days to allow the bats to adapt to the final implant weight.

#### Insertion of Neuropixels 1.0 probes

Before probe insertion, each Neuropixels 1.0 probe was sharpened at a 20°–30° angle for 15 min using a Microgrinder (EG-45; Narishige), and a single stainless-steel wire (203.2 μm coated; A-M Systems) was soldered to the ground and reference of the probe. The probe insertion procedure follows the same general surgical practice as described above. In brief, bats were anaesthetized and placed in a stereotaxic apparatus. The training cone was removed and up to three probes were inserted into pre-existing craniotomies after a durotomy. After mounting the Neuropixels 1.0 probe on a stereotaxic arm, the probe shank was coated with fluorescent dye (CM-DiI; Invitrogen C7001) and inserted into the target craniotomy at a rate of around 10–20 μm s^−1^ to a depth of 5,500 μm. The probe was then cemented in place using dental acrylic. When the cement had fully cured, the ground wire of the probe was connected to the pre-existing ground screw. After all probes had been inserted, a new 3D-printed cone was positioned and cemented to the skull. The ribbon cable of each probe was then connected to a connector piece (SpikeGadgets) attached to the top of the cone, which serves both as a protective cap and as the interface between the Neuropixels 1.0 probes and the wireless headstage. The bat was then woken up using reversal agents to counteract the dexmedetomidine and midazolam.

### Electrophysiology data acquisition, preprocessing and spike sorting

Recordings began one day after probe insertion and were performed using a SpikeGadgets wireless Neuropixels 1.0 headstage, which was attached to the connector piece on the implanted cone (along with a battery and SD card) before each recording session. The maximum number of recordable channels was 384 in total, from up to 3 probes. Channel selection to target the hippocampal pyramidal layers (dorsal CA1 and CA3) was determined by detecting high-frequency ‘ripples’ in the LFP signal together with a transient (50–100 ms) increase in multi-unit activity, monitored during a dedicated rest session before the start of the experiments. Electrical signals (referenced to the ground screw) in the spike band (600–6,000 Hz) and LFP band (0.5–200 Hz) were amplified 500–1,000× and 125–250×, respectively, and were logged locally to a SD card on the headstage. After each recording session, the headstage was removed and the SD card was retrieved. Recorded data on the SD card were downloaded using a logger dock (SpikeGadgets). Drift correction and spike sorting were done automatically using Kilosort4^53^. All units labelled by Kilosort4 as ‘good’ were kept, after visual examination in Phy^54^. Duplicated cells on the same contact (with peak cross-correlation within 5 ms) were merged and spikes removed if closer than 1 ms.

### Histology

At the end of the experimental sessions, bats were given a lethal overdose of sodium pentobarbital and perfused transcardially (200 ml phosphate-buffered saline (PBS), 0.025 M, pH = 7.4; 200 ml of fixative, 3.7% formaldehyde in PBS). After perfusion, the probe implant was carefully removed, and the brain was dissected and stored in the fixative solution for one to two days. The fixed brain was subsequently moved to a 30% sucrose solution in PBS overnight for cryoprotection, and 40-µm coronal sections were cut using a microtome (HM450; Thermo Fisher Scientific) with a freezing stage. Slices around the dorsal hippocampus and including the implant were stained for DAPI (Thermo Fisher Scientific) and cover-slipped with aqueous mounting medium (ProLong Gold Antifade Mountant, Thermo Fisher Scientific). Fluorescent images of each section surrounding the implant were acquired using an Axioscan Slide Scanner (Zeiss), and used to localize Neuropixels probe tracks, visualized from CM-DiI fluorescence. Probe positions were determined by serial reconstruction from adjacent coronal sections. All probes were successfully identified in the dorsal hippocampus of implanted bats.

### LFP recordings with cerebellar ground

Two bats were used to examine LFPs referenced to a cerebellar ground screw (*n* = 1 bat implanted with 2 Neuropixels probes; *n* = 1 bat implanted with 1 Neuropixels probe; 5 sessions each). Experiments were performed as described above. On a subset of the sessions, bats were encouraged to fly by a human experimenter in the room, to ensure sufficient spatial movement for evaluation of LFP during flight.

### Recording and detection of echolocation calls

Recording and detection of echolocation calls was done as described previously^14^ (*n* = 4 bats, indoor flight enclosure). In brief, a dedicated ultrasonic microphone (M50; Earthworks) was used to record sounds inside the experimental flight room. The microphone was connected to a preamplifier (OctaMic II; RME Synthax) and recorded audio data at a 192-kHz sampling rate. Audio recordings were controlled with the SoundMexPro (HorTech) toolbox for MATLAB (MathWorks) and recorded using custom MATLAB scripts. For detecting echolocation calls, down-sampled audio data (96 kHz) were bandpass-filtered (10–40 kHz) and *z*-scored. All events larger than 10 standard deviations were considered as potential echolocation clicks and identified with the MATLAB function findpeaks, with a minimum peak distance of 10 ms. Echolocation calls were then identified as the most abundant cluster in the space defined by the first three principal components of the power spectrum of all putative clicks (*k*-means). The correspondence between this cluster and actual echolocation clicks was confirmed by the presence of two prominent peaks in the inter-click-interval distribution, in line with what is expected for this species^55^, and by the prominent phase relationship of echolocation clicks with the wing-beat signal.

### Data analysis

All analyses were done using custom code in MATLAB (2021a, MathWorks).

### Processing positional data during behaviour

Positional data recorded by the marker-based (120-Hz acquisition frequency; four bats) or RTLS-based (100-Hz acquisition frequency; two bats) systems were preprocessed^13,14^, to obtain continuous and smooth 3D positional data. Data from the RTLS system were resampled from 100 Hz to 120 Hz, to allow for shared downstream analysis. Flights were identified on the basis of a velocity threshold of 0.5 ms^−1^, and used to segment a bat’s session into rest and flight epochs. Three-dimensional spatial trajectories during flight were clustered into similar paths using hierarchical clustering^13,14^. In brief, flight trajectories were spatially down-sampled to seven points per flight (the first and last points corresponded to the take-off and landing positions, respectively). The Frechet distance^56^ between down-sampled flights was used as a measure of flight similarity and similar flights were clustered by agglomerative hierarchical clustering. The linkage distance was set to 0.6–1.5 m after manual inspection of flight groupings. The resulting clusters consisted of highly similar flight paths and were used for all downstream analysis, excluding trajectories with fewer than five flights per cluster. Turns were identified as moments of high curvature in the middle of a flight, by finding the maximum of the smoothed 3D curvature (Gaussian kernel: 0.12 s). Flight tails (below 25% and above 75% trajectory length) were forced to a curvature of 0 m^−1^ to avoid edge effects. On the basis of the value of maximum curvature across the dataset, flight trajectories were classified into straight flights (max curvature < 1 m^−1^) versus loops (max curvature > 1 m^−1^). Absolute deviation from *g* for quantifying movement level was calculated as the absolute value of the magnitude of the accelerometer signal minus the gravitational acceleration *g*.

### LFP processing

LFPs from all recorded channels (384 channels across 2 or 3 probes) were collected at a 2.5-kHz acquisition frequency and down-sampled to 500 Hz for downstream processing. For detecting SWRs, one probe was selected after visual examination of the signal. Next, one channel was selected for every pair of collinear recording sites (the one with highest root mean squared (RMS) signal) and all the resulting channels were processed for SWR detection. In brief, the LFP signal of each channel was bandpass-filtered (100–200 Hz, stopband attenuation of 60 dB), and the ripple power was calculated as the absolute value of the Hilbert transform and smoothed with a 50-ms Gaussian kernel. Peaks in the *z*-scored ripple power exceeding a value of 3 were detected, with a minimum peak distance of 50 ms and a minimum peak width of 10 ms, after excluding flight epochs. Candidate events simultaneously detected across channels were merged, when closer than 50 ms, keeping only the one with the largest ripple power. The correlation between the signal across channels of each candidate event and the average of all events was calculated, and only events with a minimum correlation value of 0.2 were kept, using the stereotyped depth profile of SWRs. Analyses of the relationship between population firing rate and SWRs (Extended Data Fig. 3) were performed on large-amplitude and well-defined events (minimum correlation: 0.3; minimum *z*-scored ripple power: 5).

For the analysis of theta oscillations, one channel from one probe was selected after visual examination of the raw LFP data and of the distribution of relative power in the theta band (4–11 Hz) across channels. The selected channel was either the one with the highest relative theta power or—when no clear peak was visible in the theta power distribution across channels—one channel around the estimated region corresponding to the hippocampal fissure–CA1 stratum lacunosum-moleculare, where the amplitude of theta is expected to be the largest^23,57^. Analysis of theta was then performed on the average LFP signal from the optimal channel and its four nearest neighbours. The resulting signal was bandpass-filtered in the theta (4–11 Hz) or delta (1–4 Hz) range; power in each frequency band was calculated as the absolute value of the Hilbert transform. Theta bouts were detected as events of minimum 1-s duration, where the ratio of theta to delta power exceeded the value of 3, after joining events closer than 100 ms. Relative power in the theta band during flight or bouts was calculated from the LFP power spectral density, obtained using the MATLAB function pwelch. A flight was considered to be associated with significant theta oscillations if the median theta-to-delta ratio during flight was higher than 2 and if there was a significant increase in theta power in the first 3 s of flight, compared with the 3 s before flight (Wilcoxon rank-sum test).

### Replay analysis: spike sequences

Analysis of replay events using spike sequences followed three steps for each flight trajectory: (1) identification of spatially tuned cells; (2) detection of candidate replays; and (3) quantification of replay-associated metrics.

#### Identification of spatially tuned cells

Ensembles of spatially tuned cells were identified for each flight trajectory as follows. Spatial firing fields along flight paths (one-dimensional (1D) fields) were calculated for each repeated path and neuron^14^. To compute the 1D fields, we linearized flight paths as 1D trajectories between take-off and landing (bin size: 0.15 m) and calculated the average firing rate, equal to the average number of spikes in a spatial bin, divided by the bin occupancy. The firing rate was smoothed with a Gaussian window (seven bins) to generate the 1D field. Spatial information (SI) per spike^23,58^ was calculated by summing across all bins:

1 $${\rm{SI}}={\sum }_{i}\frac{{p}_{i}{\lambda }_{i}}{\lambda }{\log }_{2}\frac{{\lambda }_{i}}{\lambda },$$

where *p*_*i*_ is the probability of being in bin *i*, *λ*_*i*_ is the firing rate on the same bin and *λ* = Σ_i _*p*_*i*_*λ*_*i*_ is the average firing rate across all bins. Stability of 1D fields within a session was measured by calculating the Spearman correlation between 1D fields for the first versus the second half of the flights. One additional quantity (pks) was calculated to avoid ambiguities in the sequence position for neurons with more than one prominent place field. pks had a value of ‘0’ for cells with one place field (no peaks larger than half of the peak firing), or it was equal to the ratio between the second and the first peak for cells with more than one prominent place field. For spike-sequence analysis, we considered only cells with more than one spike per flight, more than 3 Hz firing at the field peak, a minimum stability of 0.4 and a maximum pks value of 0.5 (defined as spatially tuned cells or place cells). The same neurons were considered for the analyses of the relationship between trajectory length and place-field size (that is, the width at half prominence) or distance between place fields (Extended Data Fig. 8).

#### Detection of candidate replays and quantification of replay metrics

Candidate replays were detected for each flight trajectory as peaks in the spike density, calculated by pooling all the spikes from spatially tuned cells and convolving them with a Gaussian kernel (100 ms). Flight times were excluded from the analysis and peaks in the spike density exceeding two standard deviations were found (minimum duration: 50 ms; maximum duration: 1 s). Events separated by less than 200 ms were joined, to make sure that discontinuous replays were not left undetected. For each candidate replay, we calculated a series of features, after sorting the cell identities on the basis of the position of their spatial responses along the 1D flight paths. Replay metrics included the number of cells participating in the event, the ratio between this number and the total number of cells active during the corresponding flight trajectory, the replay duration (corresponding to the width of the spike-density event) and the rank correlation between the order of first spike and time. A *P* value was assigned to each replay by comparing the observed rank correlation with the rank correlations obtained from a shuffled distribution, in which cell identities were randomly permuted 100 times^4^. The *P* value was calculated as the fraction of shuffles with a rank correlation greater (in absolute value) than the observed one. Replays were considered good if they had a rank correlation greater than 0.2 (absolute value), a minimum number of active cells greater than 5, a minimum of 30% of the spatially tuned cells were active during the replay and the *P* value was smaller than 0.05. All replays occurring during rest and meeting quality criteria were included in the analysis. For the analysis of the relationship between replay and behaviour (Extended Data Fig. 5), flights were categorized into trajectories to feeder when the bat landed within 75 cm of the feeder. In addition, replays were categorized as: (1) immediate previous, replaying the trajectory of the most recent flight; (2) immediate next, replaying the trajectory of the upcoming flight; or (3) other, replaying a trajectory that was that of neither the previous nor the next flight. A small fraction of replays (average 2%; *n* = 23 sessions from 6 bats) was for trajectories that overlapped with both the previous and the next flights (that is, loops with shared take-off and landing sites) and was excluded from subsequent quantifications to preserve statistical power in multiple comparisons. To ensure robust categorization, we also excluded a small subset of replays (5.4%) that occurred before the first or after the last flight in a session, and only included trajectories that were replayed more than ten times. Chance levels were calculated for each session as one over the number of different flight paths that could be replayed. For the analysis of the relationship between replay duration and flight duration (Fig. 2), we focused on the subset of forward replays with a minimum of 0.4 rank correlation, 7 active cells, 30% of spatially tuned cells and *P* < 0.05. Replay speed from spike sequences was calculated as the number of neurons per second during replay, multiplied by the average metres per neuron. Neurons per second was obtained by fitting a line to the times of the first spike during replays, and metres per neuron was calculated as the distance between the last and the first place cells divided by the number of place cells in the trajectory.

### Replay analysis: decoding

#### Spatial response calculation and Bayesian decoder

Replay detection through spike-sequence analysis (see above) is based on thresholding spike density from place cells and is therefore biased towards events that involve large numbers of place cells and/or large firing rates. Given that replay-like events during immobility periods can happen in the absence of population bursts^59^, we implemented a complementary method for detecting replays, using a continuous replay detection procedure that does not rely on SWR events or population bursts. The same procedure, based on Bayesian decoding was repeated using spatial responses from each flight trajectory type.

In brief, after categorizing flights into clusters, the position of the bat during each flight was linearized from take-off to landing and the entire trajectory was divided into 30 position bins. Spatial responses of each cell were calculated as the number of spikes fired in a particular position bin divided by the occupancy of the position bin, smoothed with a Gaussian kernel with a standard deviation of two bins. Posterior probability for each linearized positional bin *x*, given the vector **n** of spikes emitted by *N* neurons at a specific time was calculated using a Bayesian decoder with uniform prior^20^:

2 $$P(x|{\bf{n}})=C(\mathop{\prod }\limits_{i=1}^{N}{f}_{i}{(x)}^{{n}_{i}}){{\rm{e}}}^{-\tau \mathop{\sum }\limits_{i=1}^{N}{f}_{i}(x)},$$

where *f*_*i*_(*x*) is the spatial response of cell *i* at positional bin *x*, *n*_*i*_ is the number of spikes emitted by cell *i*, *τ* is the size of the time bin and *C* is a normalization constant that can be determined by imposing unitary sum of *P*(*x|***n**) at each time bin. To enhance the smoothness of the decoded probability, each spike was mirrored by ±5 ms before calculating the vector **n**. All subsequent analyses, including shuffling, used the sequence of real plus mirrored spikes as input. For validation of decoding during flight, the decoder was applied on 50-ms non-overlapping time windows during flight time and a normalized root mean squared error was calculated from the estimated bat position (spatial bin with maximum posterior probability), minus the observed bat position, and normalized by the duration of the flight. For replay detection, the Bayesian decoder was applied to spikes within a 20-ms sliding window, shifted by 5-ms increments over the entire session excluding flight epochs. Different metrics were used to assess the quality and significance of replay events, and are described below.

#### Metrics for evaluating decoded events

Each candidate event was assigned four scores^20,21,59,60^ (weighted correlation, replay score, posterior spread and trajectory coverage). Each decoded event could be graphed as a two-dimensional matrix of probabilities, with time on the *x* axis and predicted position on the *y* axis. The predicted position at each time bin was defined as the position bin with the highest decoded probability. Weighted correlation was defined as the Pearson’s correlation between time and predicted position, weighted by the posterior probability of the decoded position^21^. Replay score^20^ was defined as the concentration of posterior probability within a line depicting an idealized linear trajectory along the entire track. To determine the idealized trajectory, we found the line of best fit for the predicted position (defined for each time bin as the position bin with the highest decoded probability). Posterior spread^59^ was defined as the square root of the second moment of the posterior. Trajectory coverage was defined as the percentage of trajectory length that was being covered by the replay. This value was found by fitting a linear line on the maximum posterior probability at each replay time bin, taking the difference in line position between the first and the last time bin, then dividing the trajectory covered with the trajectory length to obtain a value between 0 (the decoded sequence is horizontal and thus unlikely to be a replay of flight) and 1 (the entire flight is being replayed).

#### Continuous detection of replay events using whole-session decoding

Time bins were excluded from further analysis if there was a low confidence of decoded position, as indicated by a posterior spread score greater than 0.3, or if the probability at the position of maximum probability is less than three times the average probability. Each remaining region of continuous time bins was defined as a subsequence. Subsequences of less than 25 ms were excluded owing to the high chance of being noisy. Considering the possibility that long subsequences might get fragmented, neighbouring subsequences were merged if the temporal gap was less than 75 ms (without removing the low-confidence gaps). Sequences that contained more than 70% of low-confidence regions were excluded. Disjoint subsequences constituted candidate replays. Next, to find the centre of the replay, each candidate event was randomly trimmed on both ends 1,000 times (>50% of the original event duration) and the weighted correlation as well as the trajectory coverage were calculated for the resulting segments. One segment from the top 5% of the sum of weighted correlation and trajectory length was selected randomly. This randomness was introduced to avoid systematic bias. Segments with a short duration (<50 ms) were excluded from subsequent analysis. After finding the segment central region, the whole candidate replay event was found by including regions of high decoded confidence on either side, stopping when a low-decoded-confidence region lasting at least 50 ms was found.

#### Shuffling

After a candidate event had been found, two shuffling methods were used to determine the significance of the event. The first was a circular shift of position within each time bin, which aimed to preserve local smoothness in position by circularly shifting decoded probabilities independently for each time bin. The second method was a time shuffle, in which the time bins of an event are shuffled. The weighted correlation and replay score of the shuffled sequence were determined. *P* values were calculated after 100 shuffles by each method, and events with significant *P* values (*P* < 0.05) for both shuffling methods and both replay metrics (weighted correlation and replay score) were kept for subsequent analysis. Replays were considered good if they had a weighted correlation score (absolute value) greater than 0.4, replay score greater than 0.4 and trajectory coverage greater than 0.5. All replays occurring during rest and meeting quality criteria were included in the analysis. For the analysis of the relationship between replay duration and flight duration, an additional restriction was that the trajectory coverage needs to be greater than 0.7 to ensure that the replays cover a significant portion of the trajectory. Replay speed was calculated by dividing the length of the flight being replayed by the duration of the replay.

### Wing-beat phase extraction, phase locking and autocorrelation function

The wing-beat frequency was calculated as the peak frequency (in the interval 6–10 Hz) of the power spectrum obtained from the magnitude of the fast Fourier transform (FFT) of the absolute acceleration (norm of the 3D accelerometer signal) during flight epochs. For a subset of the analyses, we also extracted a time-varying instantaneous wing-beat frequency using the Hilbert transform of the absolute acceleration (MATLAB function instfreq), bandpass-filtered between 7 Hz and 9 Hz. Note that, as expected, the FFT estimate closely matched the time average of the instantaneous one (Pearson’s *c* = 0.83, *P* = 0, *n* = 1,442 flights from 6 bats; Extended Data Fig. 10a). The wing-beat phase was calculated as the phase of the Hilbert transform of the bat’s absolute acceleration, filtered between 7 Hz and 9 Hz. Phase 0 was defined as the trough of the absolute acceleration, such that the wing downstroke corresponded to phase 0 − π. The wing-beat phase of each spike emitted during flight was calculated as the phase of the closest wing-beat sample (as accelerometer data was acquired at 500 Hz). For each cell, a spike phase distribution was calculated by binning phase values into 20 angular bins between −π and +π. Phase distributions were cloned with a 2*π* shift for visualization and fitting. Fitting normalized phase distributions between −1 and 1 was done using a cosine function cos(*ax* − *b*) and *b* > 0. Spatially tuned cells with an *R*^2^ (coefficient of determination) greater than 0 and a minimum of 50 spikes during flight were considered as phase locked and were used to determine population phase locking, by averaging their phase distributions. The resulting average phase correlation was corrected for uneven wing-beat phase distribution by subtracting the wing-beat phase distribution, averaged across the same cells. Phase locking was also examined using standard circular statistics measures. The mean resultant vector length *r* was defined as:

3 $$r=\left|\frac{1}{n}\mathop{\sum }\limits_{j=1}^{n}{{\rm{e}}}^{i{\theta }_{j}}\right|,$$

where *n* is the total number of spikes emitted during flight and *θ*_*j*_ is the phase of the *j*-th spike. The Rayleigh statistic is *z* = *r*^2^*n*. The significance of phase locking was assessed using a shuffling procedure, in which the mean resultant vector length of each neuron was compared with a shuffled distribution of mean resultant vector lengths obtained by randomly assigning to each spike the wing-beat phase taken from flight epochs and repeating this procedure 100 times. Similar results were obtained using the Hodges-Ajne omnibus test (average 12% phase-locked spatially tuned neurons; *r* = 0.13 ± 0.01, *z* = 8.4 ± 2.4, spike count = 718 ± 65, mean ± s.e.m., *n* = 22 sessions from 6 bats), which evaluates general deviations from circular uniformity even in small samples (*n* ≥ 30) without assuming a specific distribution (in contrast to the Rayleigh test, which assumes a unimodal von Mises distribution^61^). All neurons with a minimum of 30 spikes were included in the analysis and a neuron was considered phase locked if *P* < 0.05.

Spike autocorrelation for each cell was calculated within ±500 ms, using 10-ms time bins and normalized by the total number of counts. Residual correlation for the population average was obtained by fitting the average autocorrelation between 100 ms and 500 ms with a mono-exponential function and subtracting this fit from it. Phase precession between wing-beat phase and position of a spike was examined by calculating the Spearman correlation between distance along the flight and the wing-beat phase of each spike, after finding the phase shift that maximized the absolute value of the correlation^23,62^. Phase-locked cells were considered phase preceding when the Spearman correlation value was negative and its *P* value was less than 0.05.

### Decoding sequences during flight and relationship with the wing-beat

Position probability distributions (Fig. 3g) during flight were obtained using Bayesian decoding. First, 1D linearized spatial responses of neurons during a flight were calculated as the average firing rate in 15-cm bins, spanning each trajectory from take-off to landing, as described in ‘Identification of spatially tuned cells’. Only neurons with stability (as defined in ‘Identification of spatially tuned cells’) greater than 0.6 were used for decoding. Spatial responses from these neurons were used to train a Bayesian decoder, as was done for replay detection. Each spike was mirrored at ±5 and ±10 ms, to enhance the smoothness of the decoded probability. Posterior probability for each of the 15-cm spatial bins was calculated on a sliding window of 30-ms duration, moved by 5 ms from take-off to landing, using equation (2). The decoded position of the bat at each time bin during flight was calculated as the centre of the spatial bin with highest posterior probability. The decoding error (as typically defined in the replay literature^36^) was calculated as the decoded position minus the real position of the bat at that time bin. For each flight, we calculated a RMS decoding error and the fraction of decoded bins (removing bins where no spikes were emitted). The average decoding error during a wing-beat cycle was calculated by averaging decoding errors on single wing-beat cycles (−π to +π) from all flights with a RMS decoding error smaller than 1.3 m and fraction decoded bins higher than 0.7. Flight tails, where the bat was at less than 0.15 or at more than 0.85 of the total flight length, were discarded. Similar criteria were used for the analysis of the average decoding error across wing-beat cycles with or without echolocation, with the difference that flight tails were included (whole-flight analysis) or only the first half of the flight length was considered (first-half analysis). The shuffled average decoding error was obtained by randomly shifting the wing-beat phase at each wing-beat cycle by up to ±60 ms, repeating this procedure 20 times and averaging the results. A *P* value for the difference between real and shuffled data was calculated at each time bin as the fraction of shuffled average decoding errors that were larger than the observed average decoding errors. To find the preferred phase of decoded sweeps, we focused on large-amplitude and clearly defined events (Fig. 3i), identified across all flights with a RMS decoding error smaller than 1.3 m and fraction decoded bins higher than 0.7. A template-matching algorithm was used to segment windows of decoded probability clearly resembling sweeps, followed by a cleaning step based on a deep neural network. In brief, the average decoding error was convolved with a Gaussian-shaped template (60 ms temporal width and 0.9 m spatial extent) and candidate events were found by thresholding the resulting trace. A preferred phase was assigned to each candidate event, corresponding to the wing-beat phase at its centre. For every candidate event, the posterior probability was extracted within a (−60, +60 ms) time interval and a (−0.9, +1.8 m) space interval and shifted by the real position of the bat at each time bin. The resulting matrixes (Fig. 3i, top) correspond to the posterior probability of the bat, corrected by its real position. To filter out noisy events, we retrained AlexNet^63^ (modified to distinguish between 2 classes: good sweeps versus noise) using 1,000 manually labelled sweeps, after balancing the number of negative and positive examples (sampling a similar number from the 1,000 manually labelled sweeps). The dataset was randomly split into training (80%) and validation (20%) subsets and the network was trained using stochastic gradient descent with momentum (SGDM) for 10 epochs, with a mini-batch size of 32, an initial learning rate of 0.0001 and shuffled data at each epoch. The power spectral density of the decoding error (Extended Data Fig. 13) was calculated from the magnitude of the FFT of the decoding error during flight, after subtracting the exponentially decaying part of the spectrum (fitting with a mono-exponential for frequencies greater than 2 Hz). The peak of the spectrum between 5 Hz and 16 Hz was used as an estimate for the sweep frequency during a flight. The instantaneous sweep frequency for consecutive sweeps was calculated for the subset of automatically detected sweeps (see above) that were separated by less than 180 ms, as the inverse of the time interval between their centres, determined by the template-matching algorithm.

### Analysis of non-oscillatory LFP power and phase-locking comparisons

The analysis of non-oscillatory LFP (Extended Data Fig. 11) followed methods described in previous work^24^. In brief, the same LFP signal that was used for analysing theta oscillations (see ‘LFP processing’) was filtered between 1 Hz and 10 Hz and used for downstream processing. To extract the cycle-by-cycle phase of the non-rhythmic LFP, we linearly interpolated the times between consecutive LFP troughs between 0° and 360°. Non-oscillatory power was calculated as the square of the absolute Hilbert transform of the filtered LFP signal. Time averages during flight versus non-flight epochs were used for comparing non-oscillatory power (Extended Data Fig. 11a). Overall duration of non-flight and flight epochs for each session was matched by randomly sampling from non-flight times a number of samples equal to flight sample size. The average decoding error during a non-oscillatory LFP cycle was calculated by averaging decoding errors (as defined above) on single LFP cycles (−π to +π), using the same inclusion criteria as for the wing-beat phase averages. Cycles with a low average non-oscillatory power (below the 25th percentile of the in-flight distribution), were discarded as described previously^24^. The average decoding error during a wing-beat cycle and the shuffled distribution were calculated as described above, with the only difference being that all wing-beat cycles coming from flights with no valid LFP cycles (because of the 25th percentile power threshold) were removed from the analysis, to ensure that cycles came from the same pool of flights. The resulting mean decoding error (aligned to wing-beat or non-oscillatory LFP phases) was phase-averaged around the centre of the cycle (−π/2 to +π/2) and normalized by subtracting the mean of the phase-averaged shuffled distribution and dividing by its standard deviation.

### Statistical analysis

No formal methods were used to predetermine sample sizes; adopted sample sizes were similar to those used in relevant previous studies. No randomization of experimental sessions and no blinding to experimental conditions were used during the analysis. All statistical comparisons were performed using two-tailed non-parametric tests (Wilcoxon rank-sum test, Wilcoxon signed-rank test, bootstrap or randomization tests) unless otherwise stated.

### Reporting summary

Further information on research design is available in the Nature Portfolio Reporting Summary linked to this article.

## Online content

Any methods, additional references, Nature Portfolio reporting summaries, source data, extended data, supplementary information, acknowledgements, peer review information; details of author contributions and competing interests; and statements of data and code availability are available at 10.1038/s41586-025-09341-z.

## Supplementary information

Reporting Summary

## Source data

Source Data Fig. 1

Source Data Fig. 2

Source Data Fig. 3

## Data Availability

The dataset from this study is available from the corresponding author on reasonable request. A demo session and associated material can be found via Zenodo at 10.5281/zenodo.15738988 (ref. ^64^). Source data are provided with this paper.

## Code availability

All analyses were performed using custom code in MATLAB (MathWorks), with the exclusion of spike sorting, which was done with Python-based Kilosort4. The code generated in the current study is available at https://github.com/kevin-qi/ripple-bat.git.
