# Supplementary material for: Replay and representation dynamics in the hippocampus of freely flying bats
Source: Nature. 2025 Jul 9;645(8082):974–80. doi: 10.1038/s41586-025-09341-z (PMC12460160; doi:10.1038/s41586-025-09341-z)
Supplement: Supplementary file 1 — Reporting Summary [file 41586_2025_9341_MOESM1_ESM.pdf]

nature portfolio

Corresponding author(s): Michael M. Yartsev

Last updated by author(s): Jun 25, 2025

Reporting Summary

Nature Portfolio wishes to improve the reproducibility of the work that we publish. This form provides structure for consistency and transparency in reporting. For further information on Nature Portfolio policies, see our Editorial Policies and the Editorial Policy Checklist.

Statistics

For all statistical analyses, confirm that the following items are present in the figure legend, table legend, main text, or Methods section.

n/a	Confirmed	
☐	☒	The exact sample size (*n*) for each experimental group/condition, given as a discrete number and unit of measurement
☐	☒	A statement on whether measurements were taken from distinct samples or whether the same sample was measured repeatedly
☐	☒	The statistical test(s) used AND whether they are one- or two-sided<br>*Only common tests should be described solely by name; describe more complex techniques in the Methods section.*
☐	☒	A description of all covariates tested
☐	☒	A description of any assumptions or corrections, such as tests of normality and adjustment for multiple comparisons
☐	☒	A full description of the statistical parameters including central tendency (e.g. means) or other basic estimates (e.g. regression coefficient) AND variation (e.g. standard deviation) or associated estimates of uncertainty (e.g. confidence intervals)
☐	☒	For null hypothesis testing, the test statistic (e.g. *F*, *t*, *r*) with confidence intervals, effect sizes, degrees of freedom and *P* value noted<br>*Give P values as exact values whenever suitable.*
☒	☐	For Bayesian analysis, information on the choice of priors and Markov chain Monte Carlo settings
☒	☐	For hierarchical and complex designs, identification of the appropriate level for tests and full reporting of outcomes
☐	☒	Estimates of effect sizes (e.g. Cohen's *d*, Pearson's *r*), indicating how they were calculated

Our web collection on statistics for biologists contains articles on many of the points above.

Software and code

Policy information about availability of computer code

Data collection	The 3D spatial positions of all bats were measured through either 16 motion-capture cameras (Raptor-12HS, Motion Analysis) or through an adaption of a commercially available RTLS system (Ciholas Inc.). Acquisition of neural data was done using a wireless neural logger (SpikeGadgets) recording from Neuropixels 1.0 probes. Echolocation calls were recorded via a dedicated ultrasonic microphone (Earthworks Inc.).
Data analysis	Custom MATLAB code was used for all data analysis (MATLAB version 2021a). Spike sorting was done with Kilosort4. The code generated in the current study is available at https://github.com/kevin-qi/ripple-bat.git.

For manuscripts utilizing custom algorithms or software that are central to the research but not yet described in published literature, software must be made available to editors and reviewers. We strongly encourage code deposition in a community repository (e.g. GitHub). See the Nature Portfolio guidelines for submitting code & software for further information.

Data

Policy information about availability of data

All manuscripts must include a data availability statement. This statement should provide the following information, where applicable:

- Accession codes, unique identifiers, or web links for publicly available datasets
- A description of any restrictions on data availability
- For clinical datasets or third party data, please ensure that the statement adheres to our policy

Data generated and analyzed in this study are available from the corresponding author upon reasonable request.

Research involving human participants, their data, or biological material

Policy information about studies with human participants or human data. See also policy information about sex, gender (identity/presentation), and sexual orientation and race, ethnicity and racism.

Reporting on sex and gender	Not applicable
Reporting on race, ethnicity, or other socially relevant groupings	Not applicable
Population characteristics	Not applicable
Recruitment	Not applicable
Ethics oversight	Not applicable

Note that full information on the approval of the study protocol must also be provided in the manuscript.

Field-specific reporting

Please select the one below that is the best fit for your research. If you are not sure, read the appropriate sections before making your selection.

☒ Life sciences ☐ Behavioural & social sciences ☐ Ecological, evolutionary & environmental sciences

For a reference copy of the document with all sections, see nature.com/documents/nr-reporting-summary-flat.pdf

Life sciences study design

All studies must disclose on these points even when the disclosure is negative.

Sample size	We analyzed a dataset of 33 sessions from 8 bats, while recording behavior and neural activity from 49-322 neurons per session. No statistics were used to determine sample size. Sample sizes are similar to those used in the field (e.g. Foster and Wilson, Nature 440, 680–683 (2006); Diba and Buzsàki, 10, 1241–1242, Nature Neuroscience (2007); Forli and Yartsev, Nature 621, 796–803 (2023) .
Data exclusions	Experimental sessions were excluded only if the bat did not fly at least 5 structured flights per session. The inclusion criteria for the neurons were based on sufficient spike number, behavioral coverage and firing stability (Methods), and are standard for this research field. Exclusion criteria were not predetermined.
Replication	Across n = 6 animals and 23 independent recording sessions (conducted across multiple days), the observed effects on neural activity and structured behavior were consistently replicated. The reported effects were found across all tested animals (Methods and Extended Data Fig. 14).
Randomization	Not relevant, as our study did not involve treatment of experimental groups and no randomization was required.
Blinding	Not relevant, as our study did not involve treatment of experimental groups and no blinding was required.

Reporting for specific materials, systems and methods

We require information from authors about some types of materials, experimental systems and methods used in many studies. Here, indicate whether each material, system or method listed is relevant to your study. If you are not sure if a list item applies to your research, read the appropriate section before selecting a response.

Materials & experimental systems

n/a	Involved in the study
☒	☐ Antibodies
☒	☐ Eukaryotic cell lines
☒	☐ Palaeontology and archaeology
☐	☒ Animals and other organisms
☒	☐ Clinical data
☒	☐ Dual use research of concern
☒	☐ Plants

Methods

n/a	Involved in the study
☒	☐ ChIP-seq
☒	☐ Flow cytometry
☒	☐ MRI-based neuroimaging

Animals and other research organisms

Policy information about studies involving animals; ARRIVE guidelines recommended for reporting animal research, and Sex and Gender in Research

Laboratory animals	Eight lab-born adult (3-6 years old) male Egyptian fruit bats (Rousettus aegyptiacus) were used in this study.
Wild animals	No wild animals were used in this study
Reporting on sex	All animals involved in this study were males.
Field-collected samples	This study did not involve samples collected from the field
Ethics oversight	All procedures performed in this study were approved by the Institutional Animal Care and Use Committee at the University of California, Berkeley, and were performed in accordance with the Guide for the Care and Use of Laboratory Animals.

Note that full information on the approval of the study protocol must also be provided in the manuscript.

Plants

Seed stocks	*Report on the source of all seed stocks or other plant material used. If applicable, state the seed stock centre and catalogue number. If plant specimens were collected from the field, describe the collection location, date and sampling procedures.*
Novel plant genotypes	*Describe the methods by which all novel plant genotypes were produced. This includes those generated by transgenic approaches, gene editing, chemical/radiation-based mutagenesis and hybridization. For transgenic lines, describe the transformation method, the number of independent lines analyzed and the generation upon which experiments were performed. For gene-edited lines, describe the editor used, the endogenous sequence targeted for editing, the targeting guide RNA sequence (if applicable) and how the editor was applied.*
Authentication	*Describe any authentication procedures for each seed stock used or novel genotype generated. Describe any experiments used to assess the effect of a mutation and, where applicable, how potential secondary effects (e.g. second site T-DNA insertions, mosiacism, off-target gene editing) were examined.*
